# Retrovirus-derived *RTL5* and *RTL6* genes are novel constituents of the innate immune system in the eutherian brain

**DOI:** 10.1242/dev.200976

**Published:** 2022-09-27

**Authors:** Masahito Irie, Johbu Itoh, Ayumi Matsuzawa, Masahito Ikawa, Hiroshi Kiyonari, Miho Kihara, Toru Suzuki, Yuichi Hiraoka, Fumitoshi Ishino, Tomoko Kaneko-Ishino

**Affiliations:** ^1^ Faculty of Nursing, School of Medicine, Tokai University, Kanagawa 259-1193, Japan; ^2^ Department of Epigenetics, Medical Research Institute (MRI), Tokyo Medical and Dental University (TMDU), Tokyo 113-8510, Japan; ^3^ Department of Pathology, School of Medicine, Tokai University, Kanagawa 259-1193, Japan; ^4^ Department of Genomic Function and Diversity, MRI, TMDU, Tokyo 113-8510, Japan; ^5^ Animal Resource Center for Infectious Diseases, Research Institute for Microbial Diseases, Osaka 565-0871, Japan; ^6^ Laboratory for Animal Resources and Genetic Engineering, RIKEN Center for Biosystems Dynamics Research, Kobe 650-0047, Japan; ^7^ Laboratory of Genome Editing for Biomedical Research, MRI, TMDU, Tokyo 113-8510, Japan; ^8^ Laboratory of Molecular Neuroscience, MRI, TMDU, Tokyo 113-8510, Japan

**Keywords:** Retrovirus-derived acquired genes, Eutherian-specific gene, Microglia, Innate immunity, Brain, Pathogen reaction

## Abstract

Retrotransposon Gag-like 5 [*RTL5*, also known as sushi-ichi-related retrotransposon homolog 8 (*SIRH8*)] and *RTL6* (also known as *SIRH3*) are eutherian-specific genes presumably derived from a retrovirus and phylogenetically related to each other. They, respectively, encode a strongly acidic and extremely basic protein, and are well conserved among the eutherians. Here, we report that *RTL5* and *RTL6* are microglial genes with roles in the front line of innate brain immune response. Venus and mCherry knock-in mice exhibited expression of RTL5-mCherry and RTL6-Venus fusion proteins in microglia and appeared as extracellular dots and granules in the central nervous system. These proteins display a rapid response to pathogens such as lipopolysaccharide (LPS), double-stranded (ds) RNA analog and non-methylated CpG DNA, acting both cooperatively and/or independently. Experiments using *Rtl6* or *Rtl5* knockout mice provided additional evidence that RTL6 and RTL5 act as factors against LPS and dsRNA, respectively, in the brain, providing the first demonstration that retrovirus-derived genes play a role in the eutherian innate immune system. Finally, we propose a model emphasizing the importance of extra-embryonic tissues as the origin site of retrovirus-derived genes.

This article has an associated ‘The people behind the papers’ interview.

## Teaser

**Highlighted Article:** *RTL5* and *RTL6* are eutherian-specific genes acquired from a retrovirus in microglia in the brain. They play important roles in pathogen clearance in the eutherian brain immune system.

## INTRODUCTION

In humans and mice, 11 retrotransposon Gag-like (RTL) genes encode proteins exhibiting ∼20-30% homology to the sushi-ichi long terminal repeat (LTR) retrotransposon GAG proteins, and in some cases also to POL proteins. As the ‘gypsy’ type of LTR retrotransposon to which the suchi-ichi retrotoransposon belongs is suggested to be an infectious retrovirus (Kim et al., 1994; Song et al., 1994), it is presumable that such RTL genes are derived from an extinct retrovirus. They exhibit a variety of biological functions in the eutherian developmental system and each protein has a unique amino acid (aa) sequence and length from 112 to 1744 aa residues (Brandt et al., 2005; Youngson et al., 2005; Ono et al., 2006; Kaneko-Ishino and Ishino, 2012, 2105). These are good examples of exaptation, i.e. gaining novel function(s) during the course of evolution, as originally proposed by Gould et al. (Gould and Vrba, 1982; Brosius and Gould, 1992). *Peg10* (also known as *Rtl2* or *Sirh1*), *Rtl1* (also known as *Peg11* or *Sirh2*) and leucine zipper, downregulated in cancer 1 (*Ldoc1*, also known as *Rtl7* or *Sirh7*) play essential but distinct roles in the formation, maintenance and endocrine regulation of the placenta in mice, respectively (Ono et al., 2006; Sekita et al., 2008; Kagami et al., 2008; Kaneko-Ishino and Ishino, 2012; Naruse et al., 2014; Kaneko-Ishino and Ishino, 2105). *Rtl1* is also involved in fetal/neonatal muscle development (Kitazawa et al., 2020) as well as in the functions of the corticospinal tract and corpus callosum, and in mammalian- and eutherian-specific brain structures (Kitazawa et al., 2021). In addition, *Rtl4* (also known as *Sirh11*) is related to cognitive function in the brain via regulation of noradrenaline (Irie et al., 2015).

Microglia originate from the extra-embryonic yolk sac in early development, migrate to the embryo and settle in the brain in the fetal stage, then ultimately propagate throughout the brain over the course of life (Ginhoux et al., 2010, 2013). Microglia are the primary innate immune cells of the brain and play a central role in the immune responses to various pathogens via a variety of Toll-like receptors (TLRs) (Hanisch and Kettenmann, 2007; Norris and Kipnis, 2018). Moreover, in the neonatal brain microglia are involved in shaping neuronal circuits during development by regulating neurogenesis. They induce filopodia formation by direct contact with neurons and phagocytose supernumerary or unneeded synapses, as well as pruning excess astrocytes in the developing amygdala (Hanisch and Kettenmann, 2007; Sierra et al., 2010; Reemst et al., 2016).

In this work, we address how *RTL6* [also known as *SIRH3* or *LDOC1-like* (*LDOC1L*)] and the phylogenetically related *RTL5* [also known as *SIRH8* or retrotransposon Gag domain like 4 (*RGAG4*)] contribute to the present day eutherian development/growth systems as eutherian-specific acquired genes, with *RTL6* being the most conserved of the RTL genes in eutherians. Importantly, both *RTL6* and *RTL5* play roles in the innate immune response in the brain against pathogens. We also discuss the importance of extra-embryonic tissues, such as the placenta and yolk sac, in which retrovirus-derived sequences are suggested to have been incubated for a long period of time, ultimately becoming novel endogenous genes by a series of selection events.

## RESULTS

### *RTL5* and *RTL6* are eutherian-specific genes encoding a strongly acidic and extremely basic protein, respectively

**Fig. 1.**
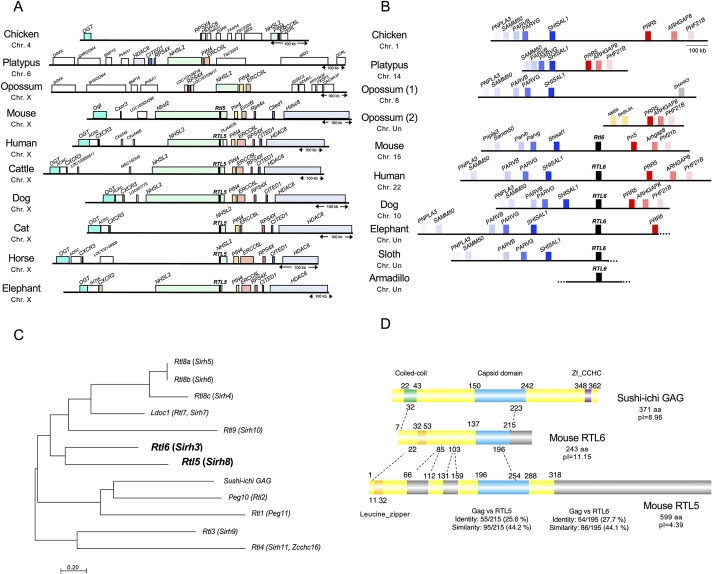
**Conservation of the *RTL5* and *RTL6* genes in eutherians and the characteristics of the RTL5 and RTL6 proteins.** (A) Chromosomal location of *RTL5* across the eutherians. The chromosomal region around *NHSL2* in which *RTL5* is located is conserved in eutherians but is slightly different in marsupials, monotremes and birds, except for *NHSL2-PIN4-ERCC6*, suggesting that chromosome rearrangements have occurred only around the *RTL5* insertion site in a lineage-specific manner. The boxes in identical colors represent orthologous genes. (B) The chromosomal location of *RTL6* is between *SHISAL1* and *PRR5*. The boxes in identical colors represent orthologous genes. In marsupials, *SHISAL1* and *PRR5* are separated into two different chromosomal loci, suggesting that chromosomal rearrangement occurred only around the *RTL6* insertion site. (C) A phylogenic tree of sushi-ichi *GAG* and all the mouse RTL genes. The scale bar indicates the number of substitutions per site. (D) Alignment of the sushi-ichi GAG (top), mouse RTL6 (middle) and RTL5 (bottom) proteins. Coiled-coil and leucine zipper motifs in the N-terminus, a capsid domain in the middle and a zinc-finger CCHC domain in the C-terminus are depicted by green and orange, blue and purple boxes, respectively. The gray boxes indicate an absence of any homology with the GAG protein.

Based on genomic data, *RTL5* is localized at the end of intron 1 of Nance-Horan syndrome like 2 (*NHSL2*) in the opposite direction (Fig. 1A and Fig. S1A), while *RTL6* lies between Shisa like 1 (*SHISAL1*) and Proline rich 5 (*PRR5*) (Fig. 1B). These sites are conserved in all four eutherian lineages, but no orthologues exist in birds, monotremes or marsupials.

Phylogenetically, *RTL5* and *RTL6* are the most closely related among the RTLs (Fig. 1C). The RTL5 and RTL6 proteins have a leucine-zipper motif in their N-terminus and exhibit a high degree of homology to each other (Fig. 1D) as well as to the suchi-ichi GAG protein (44.2 and 44.1% similarity and 25.6 and 27.7% identity, respectively). The mouse RTL5 protein, which comprises 599 aa, is strongly acidic (pI=4.39), while the mouse RTL6 protein, which comprises 243 aa, is extremely basic (pI=11.15) (Fig. S2). *RTL5* is evolutionarily well conserved in eutherians (dN/dS ratio=0.3∼0.5) (Table 1, top), but may be functionally inactive in some species due to a variety of mutations (Fig. S3, left). In contrast, *RTL6* exhibits an extremely low dN/dS ratio (mostly<0.05) across eutherian species (Table 1, bottom), indicating that *RTL6* has been subjected to very strong purifying selection (Fig. S3, right).

**Table 1.**
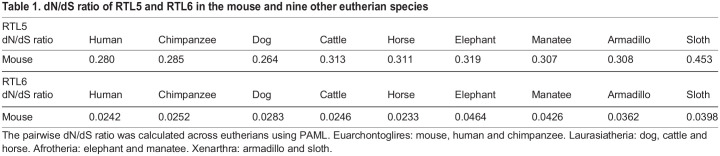
dN/dS ratio of RTL5 and RTL6 in the mouse and nine other eutherian species

### RTL6 protein expression *in vivo*

**Fig. 2.**
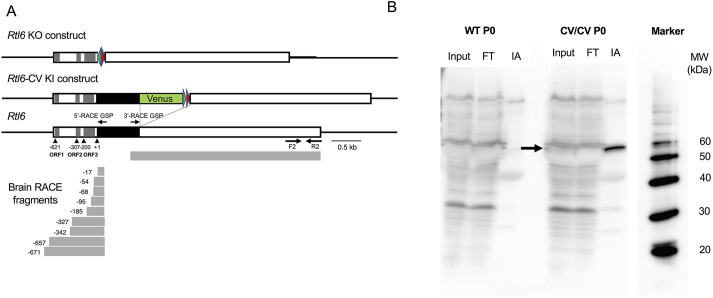
**Confirmation of RTL6 protein expression by RTL6-Venus fusion protein.** (A) The conserved mouse RTL6 ORF is encoded in the fourth ATG in the Refseq (the third line). The 5′- and 3′-RACE fragments (gray boxes) are presented below that. The four black triangles represent ATG codon sites. The construction of the *Rtl6* knockout (the first line) and *Rtl6-Venus* knock-in mouse (the second line) are also presented. Red and green triangles represent loxP and Frt sites, respectively. (B) Expression of the RTL6-Venus fusion protein in neonatal brain of *Rtl6*-CV knock-in mice. Immunoaffinity experiment. Input: total extract of P0 brain. FT, the flow through fraction; IA, the elution fraction from anti-Venus antibody beads; MW, molecular weight marker. The RTL6-CV proteins are indicated with an arrow. The RTL6-Venus protein was detected only in the *Rtl6*-CV samples (right) and not in the wild-type control (left).

We first focused on the characterization of *Rtl6* in mice because, despite the extremely conserved nature of the RTL6 protein, mouse RTL6 is encoded in the fourth open reading frame (ORF) in a reference sequence (RefSeq) of the *Rtl6* transcript in GenBank (Fig. 2A, the top panel, the third line). This strongly suggests that expression of mouse RTL6 protein is very low, even if clearly expressed, because the existence of an upstream ORF generally reduces the translational efficiency of downstream ORFs by a variety of mechanisms, such as prevention of the re-initiation of ribosomes, stalling of encoded peptides and destabilization of mRNA via nonsense-mediated decay (Calvo et al., 2009; Hinnebusch et al., 2016). Therefore, there may be a significant discrepancy between the expression levels of the *Rtl6* mRNA and RTL6 protein (see next section). In fact, it has proven to be very difficult to detect the mouse RTL6 protein by the usual methods, such as western blotting or immunostaining analysis, using either commercially available anti-RTL6 antibodies or those of our own making.

A 5′-RACE experiment on the 8-week adult brain using gene-specific primers (GSPs) from inside the protein-coding region demonstrated that among nine different 5′-RACE fragments, five of them, starting at the −185, −95, −68, −54 and −17 nucleotide positions from ATG (+1), respectively, have RTL6 as the first ORF, suggesting that the RTL6 ORF can be expressed from very short mRNAs (Fig. 2A, bottom). We then generated Venus KI mice in which a Venus ORF is integrated into the endogenous *Rtl6* locus immediately after its C-terminus (*Rtl6*-CV strain) to detect the mouse RTL6 protein *in vivo* in the same tissues and organs as the endogenous RTL6 protein (Fig. 2A, the top panel, the second line). The RTL6-Venus fusion protein was detected with immunoaffinity (IA) chromatography using an anti-Venus (GFP) antibody at the expected molecular weight of 54 kDa (27 kDa each for the RTL6 and Venus protein) in the neonatal day 0 (P0) KI mouse brain (Fig. 2B), demonstrating that it was evidently expressed in the brain.

### RTL6 expression in microglia in the central nervus system

**Fig. 3.**
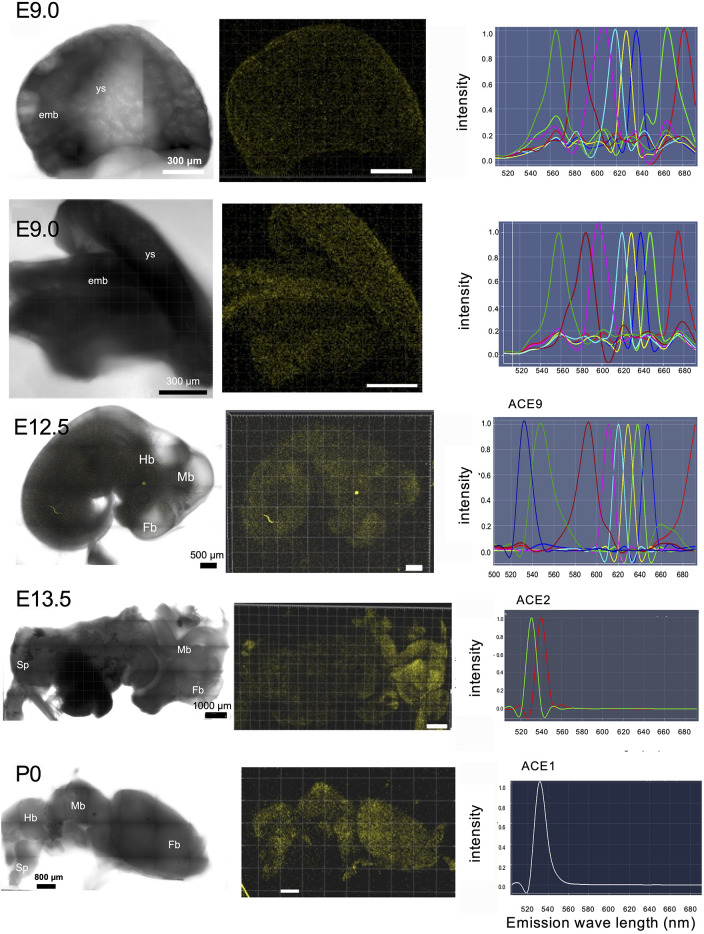
**RTL6-CV protein expression during mouse development.** RTL6-Venus signals were detected from the early developmental stage to the neonatal stages. Left, transmission image; middle, Venus signals (530 nm); right, ACE spectrum profiles; top and second rows, E9.0 embryo and yolk sac. The Venus signal was detected in both embryos and yolk but was very weak (out of ACE8, *n*>5). Third and fourth rows: the Venus signal was still very weak (ACE9) in the E11.5 embryo (*n*>3) but became strong in the CNS in the E13.5 embryo (ACE2) (*n*>3). Bottom row: P0 brain exhibited the strongest Venus signal (ACE1) (*n*>3).

The RTL6-Venus protein is expressed in microglia, and is also present as extracellular dots as well as granules in the brain. To obtain the precise position as well as the relative fluorescent strength using confocal fluorescence microscopy, it is essential that the target Venus signal (emission peak at 530 nm) be separated from various kinds of autofluorescence (Af) in the embryo, brain and other tissues (Fig. 3). Therefore, the data processing function known as multi-channel unmixing or automatic composition extraction (ACE) was applied. Confocal fluorescence microscopy analysis of the *Rtl6*-CV mice demonstrated that its expression was observed to be weak in the yolk sac and embryo on embryonic day 9.0 (E9.0) [weaker than the 8th strongest signal (ACE8)] and then became very strong (ACE2) in the central nervus system (CNS) by E13.5, with its highest expression observed in the perinatal period and postnatal day 0∼3 in the brain (P0∼3, ACE1∼2) (Fig. 3, bottom). It is thus concluded that the RTL6 protein is predominantly expressed in the CNS in mice. Even though the qPCR experiment demonstrated that brain *Rtl6* mRNA expression was higher in 4- and 8-week mice than in neonates (P0) (Fig. S4A), the RTL6 signal in the former seemed to be lower than the latter (Fig. 3 and Fig. S4B). Similarly, *Rtl6* mRNA expression was higher in the muscle, kidney and testis in 4- and 8-week adults than in the P0 brain (Fig. S4A), yet their RTL6 signals seemed lower than in the P0 brain (Fig. S4B).

**Fig. 4.**
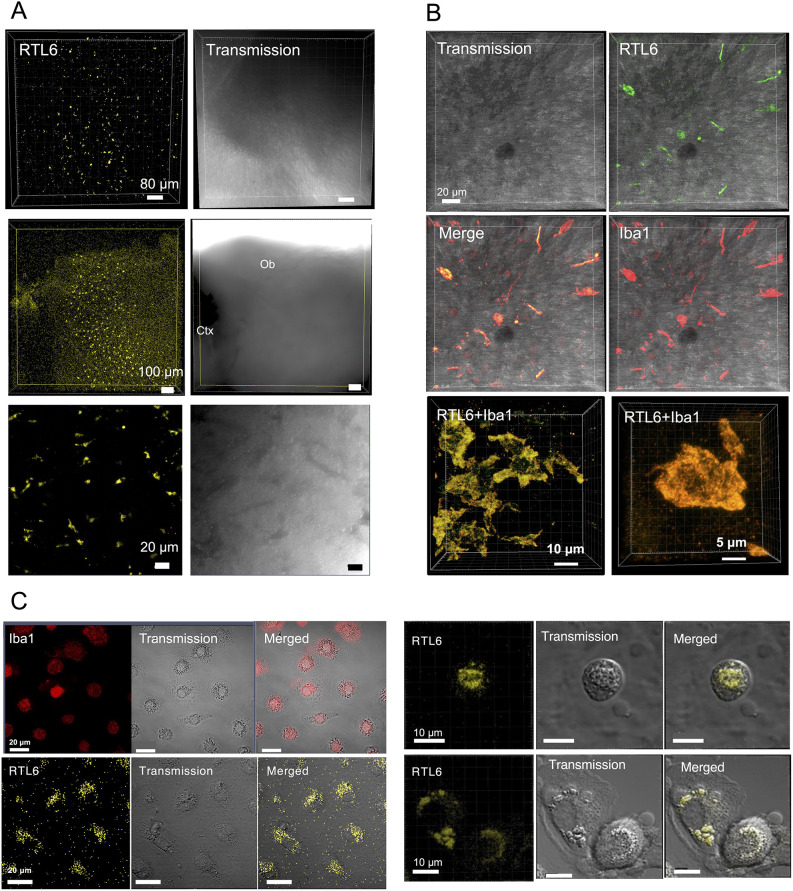
**RTL6 expression in microglia.** (A) Venus-positive cells in the hypothalamus (top), olfactory bulb (middle) and enlarged images of the olfactory bulb region (bottom). Ctx, cerebral cortex; Ob, olfactory bulb. (B) Iba1 staining of the Venus-positive cells in the hypothalamus (top and middle) and enlarged images of the Venus-positive cells (bottom). (C) Isolated microglial cells from the P1 neonatal brain. Left: a population of microglia, Iba1 staining (top) and RTL6 expression (bottom). Right: enlarged pictures of microglia showing RTL6 dots and granules in their cytoplasm.

Venus-positive cells were frequently observed in the hypothalamus and olfactory bulb regions on the internal surface of the brain in neonates (Fig. 4A,B). They were completely merged with cells expressing the microglial marker anti-ionized calcium-binding adapter molecule 1(Iba1) (Utans et al., 1995; Ito et al., 1998) (Fig. 4B), demonstrating that the RTL6-Venus protein is expressed exclusively in microglia with a variety of morphologies (round, ameboid, irregular and elongated), together with various processes (Hanisch and Kettenmann, 2007; Norris and Kipnis, 2018). In contrast, in internal regions such as the hippocampus (Fig. S5A-D) and amygdala (Fig. S5E), most of the Venus signals were spread out as small extracellular dots (much less than 1 μm in diameter), while Venus-positive cells were rarely observed. In the hippocampus, the small Venus-positive dots were accumulated alongside the pyramidal and granule cells within the hippocampal sub-regions 1-3 (CA1-CA3) (Fig. S5A) and dentate gyrus (DG) (Fig. S5B), suggesting that the RTL6-Venus protein plays some role in the region around the dendrites of these neurons (Fig. S5C), whereas only a small number of Venus-positive cells were detected (Fig. S5D). In addition, larger extracellular granules (1∼3 μm in diameter), each comprising small extracellular dots, were frequently observed in the amygdala and in some neonatal midbrain regions, such as the inferior colliculus and substantia nigra (Fig. S5E).

Finally, we isolated microglial cells from the P1 neonatal KI brain and cultured them *in vitro* (Floden and Combs, 2007; Lian et al., 2016). The cells that were collected from the cultured Petri dishes by tapping were Iba1 positive and had a round shape or an amoeba-like morphology (Fig. 4C, top in left columns). More than 40% of the P1 microglial cells expressed the RTL6-CV protein at a relatively high level (Fig. 4C, bottom in left columns) as intracellular dots and granules (Fig. 4C, right columns), similar to the Venus-positive extracellular dots and granules in the brain.

### RTL5 is also expressed in microglia

**Fig. 5.**
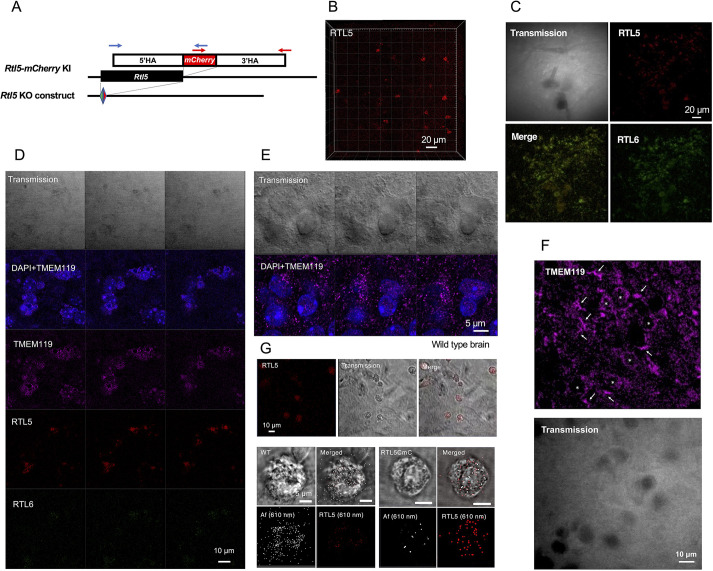
**RTL5 expression in microglia.** (A) The construction of the *Rtl5-mCherry* KI (top and middle) and *Rtl5* KO mice (bottom). (B,C) mCherry-positive cells in the olfactory bulb and hypothalamus (see also Fig. S6A-C). (D) RTL5 expression in TMEM119-positive round microglia. RTL5 were detected in some of the round type of microglia at a higher level, while the RTL6 level was relatively low. (E) TMEM119 immunostaining in the wild-type brain. A sequence of three photographs at 0.5 μm intervals is presented. (F) TMEM119 immunostaining in the DKI brain. Both the ramified (arrows) and round (asterisks) microglia were stained using the anti-TMEM119 antibody. (G) Isolated microglial cells from P1 neonatal brain of the wild-type (bottom left four images) and *Rtl5-mCherry* KI (bottom right four images) mice. Top: a population of RTL5-positive microglia (see also wild-type control in Fig. S6D). Middle and bottom rows: wild-type microglia (top left) and RTL5-positive microglia (bottom right). Af610nm- and mCherry-derived signals were present (see also Table 2).

Like RTL6, RTL5 was also found to be expressed in microglia. Using *Rtl5*-mCherry KI (*Rtl5*-CmC) mice (Fig. 5A) and *Rtl6*-Venus and *Rtl5*-mCherry double KI (DKI) mice generated by mating these strains, RTL5-expressing cells were also frequently observed in the olfactory bulb and hypothalamus on the inner surface of the hemispheres (Fig. 5B,C and Fig. S6A-C). The RTL5 protein was present as intracellular granules in round cells in the olfactory bulb (Fig. S6C, top) and also detected in the round cells in the cerebral cortex, cerebellum and midbrain (Fig. S6C, middle and bottom). In the hypothalamus, RTL5 co-existed with RTL6 in the same cells but their relative amounts seemed to vary, presumably depending on the cell (Fig. 5C). Importantly, RTL5 expression was relatively higher in the round type of microglia (Fig. 5D and Fig. S7A) that express TMEM119, another microglia-specific marker (Fig. 5E,F) (Bennett et al., 2016), and appeared to be much lower than RTL6 in the Iba1-positive ramified microglia (Fig. S7B) and elongated microglia (Fig. S7C). These results indicate that the morphologically different types of microglia express RTL5 and RTL6 at different levels and presumably play different roles in the brain.

**Table 2.**
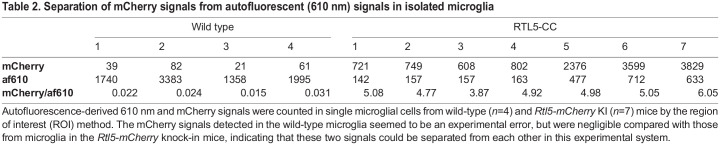
Separation of mCherry signals from autofluorescent (610 nm) signals in isolated microglia

We also confirmed RTL5 expression in isolated and cultured microglial cells (Fig. 5G). It should be noted that the autofluorescence that peaked at 610 nm was frequently detected in many tissues and organs (also in microglia), but the mCherry (610 nm) signal was usually distinguishable by using the ACE system (Fig. 5G, Fig. S6A,B,D and Table 2).

### Function of RTL5 and RTL6 against certain pathogen

RTL6 and RTL5 quickly reacted to certain pathogens, including lipopolysaccharide (LPS), dsRNA and non-methylated DNA. They formed different RTL-pathogen complexes in microglia depending on the pathogens used. Microglia are the primary innate immune cells in the brain and play a central role in the immune responses mounted to various pathogens via a variety of Toll-like receptors (TLRs) (Fiebich et al., 2018). Therefore, we analyzed the response of RTL6 and RTL5 proteins to pathogens in the brain.

**Fig. 6.**
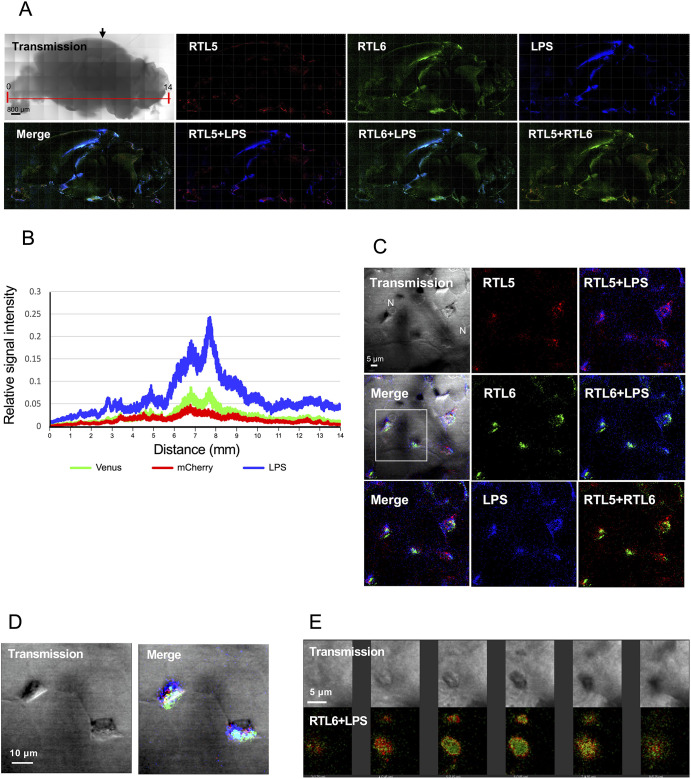
**The differential responses of RTL5 and RTL6 to LPS.** (A) The results of the LPS injections (*n*=4). RTL5 (red), RTL6 (green) and LPS (blue) signals on the inner face of the brain hemispheres (5 weeks). All of the fluorescent signals were artificially intensified, but the RTL5 signal was hardly detectable at the lower magnification. The LPS injection site is indicated by an arrow. (B) Relative intensity of each RTL5-mCherry (red), RTL6-Venus (green) and LPS (blue) signal/transmission signal along the *x*-axis (direction from the olfactory bulb to the cerebellum, which is indicated with a red line in A). (C) Giant flattened cells along with the blood capillaries in the cerebral cortex (see also Fig. S8). (D) Enlargements of the area outlined in C indicating large RTL5/RTL6/LPS complexes. (E) Spatial distribution of RTL6 and LPS in the large complexes at an intersection of giant flattened cells. Two sets of a sequence of photographs at 0.7 μm intervals are presented. Transmission (top) and fluorescent (images) images. N, nucleus. Venus (green) and Alex594-labelled LPS (red) signals are shown.

Five to 10 min after the injection of Alexa 594-labeled-LPS (emission peak 617 nm), fresh brain was dissected and directly examined under confocal fluorescence microscopy for ∼1-2 h or after fixation with paraformaldehyde (PFA). At lower magnification, the RTL6 (green) signal was observed to have accumulated in the LPS-injected regions (shown in artificial blue) (Fig. 6A). Despite a methodological limitation in obtaining the absolute value of each signal intensity, we were able to calculate the relative intensity of each signal in the whole brain from the left (olfactory bulb) to right sides (cerebellum) (Fig. 6A, the red line in the top left panel), indicating that mainly RTL6 and, to a lesser extent, RTL5 had accumulated at the LPS-injected regions in proportion to the amount of LPS (Fig. 6B). At higher magnification, the RTL5- and RTL6-expressing cells that had accumulated near the blood capillaries in the cerebral cortex had transformed into giant flattened cells and formed a barrier-like structure along with the blood capillaries by assembling together (Fig. 6C). It is known that bacterial infections and/or inflammation induce similar multinuclear giant cells (MNGCs) comprising microglia (Peterson et al., 1996; Hornik et al., 2014), suggesting that these giant flattened cells are microglia, although they seemed not to have fused, as cellular boundaries were evident (Fig. 6C,D). LPS was incorporated along the cellular edges where RTL6 had accumulated on the cytoplasmic side of the flattened microglia (Fig. S8). In addition, a large RTL5/RTL6/LPS complex was frequently observed, presumably at the intersection between three or four giant flattened cells (Fig. 6C,D). A sequence of photographs at 0.7 μm intervals indicated that RTL6 was present in the complex core and LPS on its surface (Fig. 6E), suggesting that the RTL6 complex has the capacity to trap LPS.

**Fig. 7.**
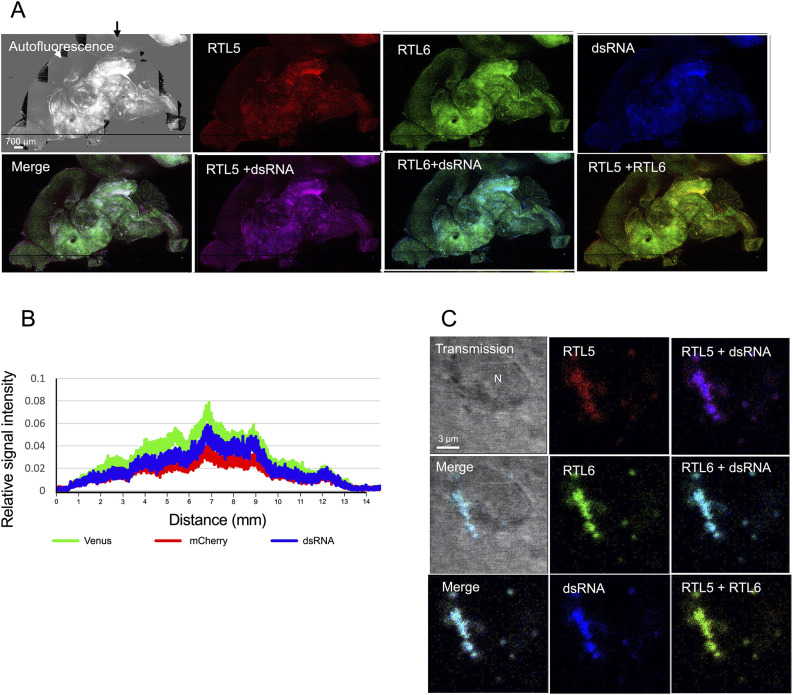
**The differential responses of RTL5 and RTL6 to dsRNA.** (A) The results for the dsRNA analogs (*n*=2). RTL5 (red), RTL6 (green) and dsRNA analog (blue) signals on the inner face of the brain hemispheres. An autofluorescence image (top left) was used instead of a transmission image in this figure because it represented the brain structure better. The dsRNA injection site is indicated by an arrow. (B) Relative intensity of each RTL5-mCherry (red), RTL6-Venus (green) and dsRNA analog (blue) signal in the P7 neonate. (C) A round cell similar to TMEM119-positive microglia with RTL5/RTL6/dsRNA complexes. N, nucleus.

Both RTL6 and RTL5 accumulated at the poly (I:C) synthetic dsRNA analog, with the injected regions (shown in artificial blue) being in proportion to the amount of rhodamine-labeled-poly(I:C) (emission peak 576 nm) (Fig. 7A,B). At higher magnification, the majority of the injected dsRNA analog accumulated in the proximity of the nucleus of round cells, similar to TMEM119-positive microglia, and formed a large, chain-like (10 μm long) complex with both the RTL6 and RTL5 proteins (Fig. 7C).

**Fig. 8.**
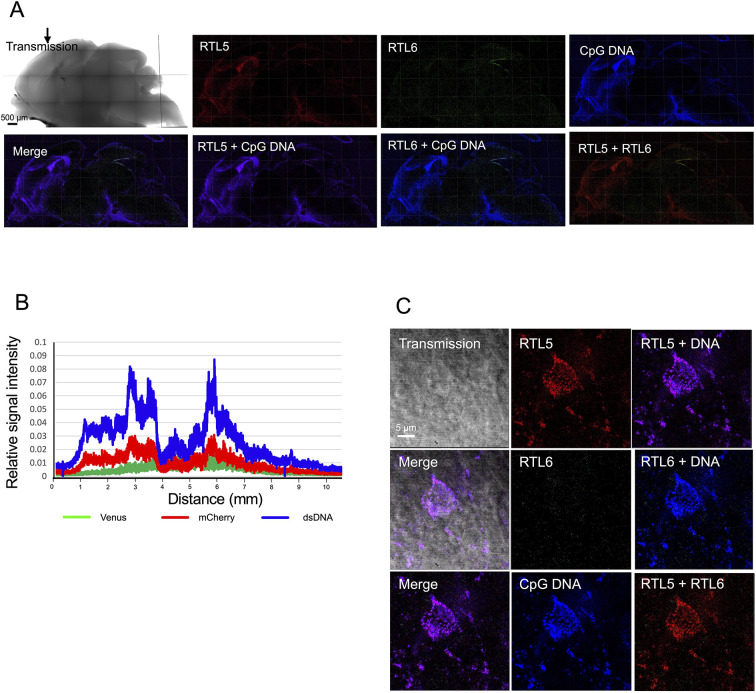
**The differential responses of RTL5 and RTL6 to dsDNA.** (A) The results for the non-methylated dsDNA (*n*=2). RTL5 (red), RTL6 (green) and dsDNA (blue) signals on the inner face of the brain hemispheres. The RTL6 signal was hardly detected in either the lower- (A) or higher-magnification (C) images. The dsRNA injection site is indicated by an arrow. (B) Relative intensity of each RTL5-mCherry signal (red). The RTL6-Venus (green) and dsDNA (blue) signal in P3 neonate. (C) A round cell similar to TMEM119-positive microglia with RTL5/dsDNA complexes accumulated near the nucleus (see also Fig. S9A).

In contrast, upon injection of non-methylated dsDNA labeled with cyanine (Cy) 3 (emission peak 570 nm, shown in artificial blue), RTL5 mainly reacted to form an RTL5/dsDNA complex (violet) without RTL6 (Fig. 8A,B). The relative signal intensity of RTL5 (red) exceeded that of RTL6, in which a higher dsDNA signal had been observed. The majority of the dsDNA was incorporated within 2 h of injection into the round cells, similar to TMEM119-positive microglia, and formed an RTL5/DNA complex without any RTL6 protein (Fig. 8C and Fig. S9A). The relative signal intensity of RTL6 usually exceeded that of RTL5 throughout the brain (Fig. S9B,C) and no reaction was observed in the control experiment using PBS except for a very subtle response at the injection sites.

### Pathogen responses in *Rtl6* KO and *Rtl5* KO mice

**Fig. 9.**
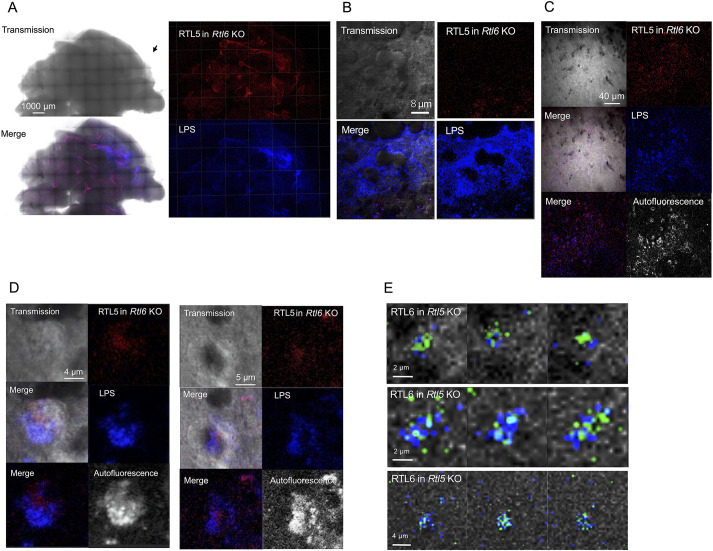
**Reduced response to LPS in *Rtl6* KO but not in *Rtl5* KO.** (A) LPS response in the *Rtl6* KO brain (*n*=4). RTL5 (red) and Alexa 488-labelled LPS (blue) signals on the inner face of the brain hemispheres. (B) There was no RTL5 accumulation in the higher LPS region but some in the lower LPS region (bottom right corner). (C) RTL5 accumulation in the lower LPS region. (D) LPS was incorporated into round RTL5-positive microglia (left and right panels). In both cases, the autofluorescence image (bottom right) partially overlapped with the LPS image (520 nm from Alexa 488) (middle right). (E) The LPS response in the *Rtl5* KO brain (*n*=2). Three sets of a sequence of photographs at 0.5 μm intervals are presented. RTL6 accumulated to LPS as granules and seemed to play a substantial role in the LPS removal in the *Rtl5* KO brain without forming the large RTL5/RTL6/LPS complex observed in the DKI brain (Fig. 6C,D).

The LPS response was significantly altered in the *Rtl6* KO mice with the *Rtl5*-mCherry insert (hereafter called *Rtl6* KO mice) compared with the *Rtl6*-Venus and *Rtl5*-mCherry DKI mice. In the *Rtl6* KO brain, the RTL5-CmC protein had partially accumulated in the LPS-Alexa 488-injected regions, although it did not completely merge with the LPS image (Fig. 9A) as it had in the DKI mice (Fig. 6A). In the regions in which the LPS signal intensity was high, much of the injected LPS remained in the extracellular space even after 2 h (Fig. 9B), indicating that LPS removal was significantly reduced in the *Rtl6* KO mice. In the lower level LPS regions, some of the RTL5-positive round cells had incorporated LPS, presumably around their nuclei (Fig. 9B, bottom right corner, Fig. 9C,D). In the case of LPS injection in the *Rtl5* KO mice, the LPS removal activity was essentially unaffected. RTL6 accumulated to LPS in the form of granules in the cytoplasm of microglia and appeared to play a crucial role in LPS removal (Fig. 9E), suggesting that RTL6 has a major role in LPS removal without the formation of RTL5/RTL6/LPS complex (Fig. 6C,D).

**Fig. 10.**
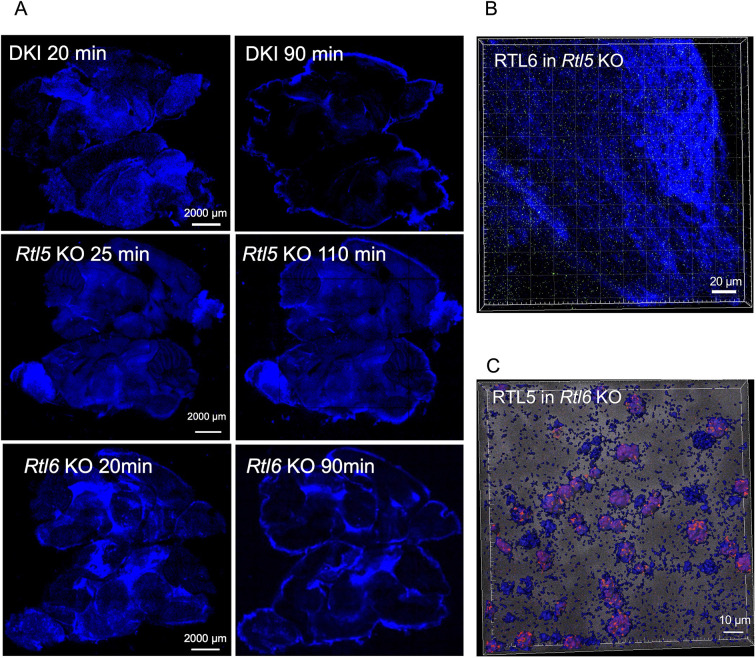
**Reduced response to dsRNA in *Rtl5* KO but not in *Rtl6* KO.** (A) Time course of dsRNA removal (*n*=3). The Poly (I:C)-Rhodamine signal (575 nm) is artificially colored in blue. The fluorescence data obtained from the same setting were compared between 20 min and 90 min after administration of the dsRNA analog (DKI and *Rtl6* KO), or 25 min and 115 min after administration (*Rtl5* KO). The signal intensity was significantly reduced in the entire brain of the DKI (top) and *Rtl6* KO (middle), whereas it remained unchanged in the *Rtl5* KO (bottom). The signal intensity around the brain increased over time, presumably owing to the signal seeping out of degraded tissues. Ten μl of 6 ng/μl dsRNA analog was administrated, and both the right and left brain hemispheres are represented in each experiment. (B) RLT6 was dispersed as small dots independently of the extracellular dsRNA analog in the *Rtl5* KO brain. (C) The dsRNA response in the *Rtl6* KO brain (*n*=2). RTL5 accumulated in the dsRNA analog as granules and seemed to play a substantial role in the dsRNA removal in the *Rtl6* KO brain without forming the chain-like RTL5/RTL6/dsRNA complex observed in the DKI brain (Fig. 7C).

In contrast, we found that the dsRNA response was greatly affected in the *Rtl5* KO mice with the *Rtl6-*Venus insert (hereafter called *Rtl5* KO mice): the signal intensity of dsRNA was significantly reduced in the DKI and *Rtl6* KO brains 90 min after administration of the dsRNA analog (Fig. 10A, top and bottom), whereas it was unchanged in the *Rtl5* KO mice and remained so even after 110 min when compared with the intensity 25 min after administration (Fig. 10A, middle), indicating that, without RTL5, dsRNA removal was significantly delayed in the brain. RTL6 was dispersed as dots independently from the distribution of dsRNA analog (Fig. 10B). In the case of dsRNA injection to *Rtl6* KO, the dsRNA removal activity seemed to be unaffected. RTL5 was accumulated in dsRNA as granules in the cytoplasm of microglia (Fig. 10C) but seemed to play a crucial role in the dsRNA removal without the formation of chain-like RTL5/RTL6/dsRNA complex (Fig. 7C). These results provide strong evidence that RTL6 and RTL5 are involved in LPS and dsRNA removal in the brain, respectively, although these observations are evidently qualitative, not quantitative. It is also possible that RTL6 and RTL5 play some role in the dsRNA and LPS removal, respectively.

## DISCUSSION

These data clearly demonstrate that *RTL5* and *RTL6* function as microglial genes in the front line of innate immunity for the quick clearance of certain pathogens, providing the first evidence that the eutherian-specific genes acquired from retroviral infection are functional in the innate immunity of the eutherian brain (Figs 4-10). Both proteins are present as intra- as well as extracellular granules in the brain, so it is possible that they act as an emergency response and immediately trap invading pathogens in order to prevent them from spreading. In these experiments, we used fresh brain samples in order to avoid an unexpected activation of microglial cells by sectioning of the fresh brain and they were examined within 2 h without fixation because the fluorescent signals decline over time and are considerably reduced after being fixed. Despite these technical limitations, the Venus and mCherry KI mice nevertheless provided clear evidence that the RTL6 and RTL5 proteins are dominantly expressed in the CNS and exclusively in brain microglia because the RTL6-Venus and RTL5-mCherry proteins would be expected to be translated in the same tissues and organs as the endogenous RTL6 and RTL5 proteins, respectively. The KI mouse experiments further demonstrated that the RTL6-Venus and RTL5-mCherry proteins respond to the LPS, dsRNA and non-methylated DNA, either cooperatively or independently. However, we cannot exclude the possibility that the Venus and mCherry tagging changes the expression level and localization of the RTL6 and RTL5 proteins to a certain extent, because, at present, there are no reliable antibodies to RTL6 and RTL5 that would allow confirmation of the findings. In addition, it is reasonable to hypothesize that the efficiency of the functions of RTL6 and RTL5 would be affected by the additional C-terminal Venus or mCherry region because their functions must have been specified in multiple evolutionary selection events over a long period of time, as discussed below.

The experiments using the *Rtl5* KO and *Rtl6* KO mice ultimately provided clear evidence that the RTL6 and RTL5 proteins play a significant role in the brain, at least in the LPS and dsRNA responses, respectively. However, it should be noted that the confocal fluorescence microscopy data in this study are qualitative, and further study using quantitative methods will be required to examine the precise pathogen removal activities as well as the interaction between RTL5 and RTL6. In addition, it will be important to evaluate the acute and chronic effects of LPS, dsRNA and non-methylated DNA administration, and/or viral infection in the *Rtl5* KO and *Rtl6* KO mice using other less invasive methods to assess their biological significance *in vivo.*

It seems very likely that, based on the retroviral GAG protein, the leucine zipper motif emerged in the N-terminus and in the extremely acidic/basic and basic C-terminus regions of RTL5 and RTL6 by multiple mutation events (Fig. 1C and Fig. S1C). According to the ColabFold prediction (AlphaFold2 using the fast homology search function of MMseqs2) (Jumper et al., 2021; Mirdita et al., 2022), the RTL6 protein possesses two long helical structures, one corresponding to the extremely basic C-terminal region and the other containing a leucine zipper motif in the N-terminus (Fig. S2, bottom and Fig. S10, right). Given that RTL6 can form dimer/oligomer structures via the leucine zipper motif, the resulting ‘bouquet-like’ structure may be able to efficiently trap acidic substances on the surface of its extremely basic helix. This would be consistent with the LPS-coated ball-like structure of the RTL6/LPS complex shown in Fig. 6E because LPS is highly acidic. ColabFold also predicts that the RTL5 protein has a strongly acidic helix (Fig. S10, left) in the long C-terminal acidic region (Fig. S2, top). Interestingly, this acidic and subsequent basic region appear spatially differentiated in the predicted 3D structure, so the RTL5 protein may efficiently bind both basic and acidic substances so as to form large complexes such as the RTL5/RTL6/LPS complex (Fig. 6C,D). Although the RTL5/RTL6/LPS and RTL5/RTL6/dsRNA complexes may not play essential roles in the LPS and dsRNA removal reactions (Figs 9E and 10C), this might be an artifact caused by Venus and/or mCherry tagging. It is reasonable to assume that the RTL5 and RTL6 proteins without the Venus and/or mCherry tagging would exhibit more rapid responses and that the actual endogenous complexes would be more efficient at pathogen removal. In order to check this possibility, we need to develop novel quantitative techniques that can be applied to non-fixed brain samples.

The dN/dS ratio of *RTL6* (<0.05, Table 1, bottom) lies between the average dN/dS ratio of the house-keeping genes (∼0.093) and the Histone H3 gene (<0.01), one of the most widely conserved genes (Kimura, 1986; Zhang and Li, 2004). This indicates that *RTL6* has been powerfully conserved in eutherians, suggesting that the role of RTL6 in LPS removal is vitally important, because LPS is an extremely dangerous pathogen. The dN/dS ratio of *RTL5* is also well conserved in eutherians (Table 1, top) despite there being some exceptions (Fig. S2), suggesting that the emergence of RTL5 has also been advantageous to the evolution of the eutherian innate immune system along with RTL6. Thus, *RTL5* and *RTL6* may be seen as precious gifts from a retrovirus. Furthermore, the eutherian-specific *RTL5* and *RTL6* are also good examples of exaptation because they work as ‘self constituents’ in the Self/Nonself discrimination system that is crucial to proper immune function.

Microglia express a variety of TLR proteins, including TLR3, TLR4 and TLR9 for dsRNA, LPS and non-methylated CpG DNA, respectively (Fiebich et al., 2018). At the moment, it remains unknown how RTL5 and RTL6 are related to the TLR system in innate immunity. As mentioned above, both proteins are present in the form of intra- as well as extracellular granules, so it is possible that they function independently of the TLR systems. For example, in the case of LPS, it is possible that pre-existing RTL5 and RTL6 proteins in the extracellular space initially react to the invading LPS before the TLR4 system responds. Alternatively, they may also act as sensors of these pathogens in the TLR systems, although our preliminary study indicated that when LPS was administrated to primary mixed glia cultures, *Il6* and *Tnfa* mRNAs induction was normal in the *Rtl6* KO microglia as there was no increment of *Rtl6* mRNA (Fig. S11). Recently, two TLR4-independent innate immune responses were reported: activation of membrane-bound transient receptor potential (TRP) channels and cytoplasmic caspase 4/5 against extra- and intracellular LPS, respectively (Kayagaki et al., 2013; Meseguer et al., 2014; Shi et al., 2014; Mazgaeen and Gurung, 2020). Therefore, redundant pathways may exist in mammals, especially against LPS. Detailed genetic and biochemical analyses of these genes and proteins will be of special interest in unraveling the uniqueness of the present day eutherian innate immune system.

In the neonatal brain, microglia are involved in shaping neuronal circuits during development via their regulation of neurogenesis. They induce filopodia formation by direct contact with neurons, phagocytose supernumerary or unneeded synapses, and prune excess astrocytes in the developing amygdala (Hanisch and Kettenmann, 2007; Sierra et al., 2010; Reemst et al., 2016). RTL5 and RTL6 thus seem likely to play an important role in maintaining a clean environment in the developing brain by removing hazardous substances leaking from damaged neuronal cells during neural network formation.

It is known that neuronal cells secrete the activity-regulated cytoskeletal (ARC) protein, a GAG-derived capsid-like substance, for communicating between and among neuronal cells via binding and delivering mRNA (Ashley et al., 2018; Pastuzyn et al., 2018). Recently, the PEG10 and RTL1 proteins were also shown to be able to form a virus-like structure with the ability to deliver mRNA, much like exosomes (Segel et al., 2021). It is very interesting to consider that the basic structure of the GAG protein has been used in a wide range of applications during the course of evolution. *ARC* was domesticated independently in tetrapods and insects (Ashley et al., 2018; Pastuzyn et al., 2018), while *PEG10* was domesticated in therians, and *RTL1*, *RTL5* and *RTL6* were domesticated in eutherians. Therefore, it is very likely that there are additional brain-related genes from other retroviral GAG sequences not only in eutherians but also in other organisms, because the domestication of such genes has proven to be of such benefit.

**Fig. 11.**
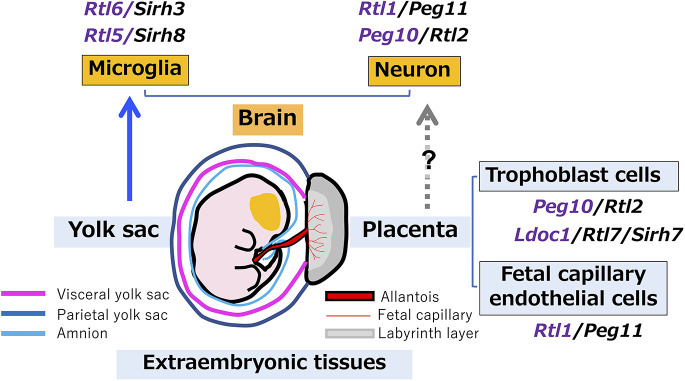
**Relationship between mammalian-specific RTL/SIRH genes and extra-embryonic tissues.** It was demonstrated in this study that *Rtl5/Sirh8* and *Rtl6/Sirh3* are expressed in yolk sac-derived microglia, and are therefore functional in the brain. To date, five out of 11 RTL*/*SIRH genes have been shown to be domesticated in extra-embryonic tissues, such as the placenta and yolk sac. Interestingly, among the three placental RTL*/*SIRH genes, *Rtl1/Peg11* is expressed and seems functional in various neurons, such as those in the corticospinal tract and corpus callosum (Kitazawa et al., 2021). *Peg10/Rtl2* is reported to be neuronally expressed in many brain regions (Chikamori et al., 2019) and the PEG10 protein in iNeurons isolated from Angelman syndrome patients (Pandya et al., 2021). It is possible that these genes were first domesticated in the placenta and then subsequently acquired brain functions during eutherian evolution.

Among the 11 RTLs, *RTL5* and *RTL6* are the first examples of genes that function in yolk sac-derived microglia (Figs 3, 4C and 5G) and have roles in the front line of brain innate immune responses against specific pathogens (Figs 6-9). Microglia originate from the extra-embryonic yolk sac in early development, migrate to the embryo and settle in the brain in the fetal stage, then ultimately propagate throughout the brain over the course of life (Ginhoux et al., 2010, 2013). We have previously demonstrated using knockout mice that *PEG10*, *RTL1* and *LDOC1* play different but essential roles in the placenta (Ono et al., 2006; Kagami et al., 2008; Sekita et al., 2008; Naruse et al., 2014), another extra-embryonic tissue. Endogenous retroviruses (ERVs) and retrotransposons are usually completely repressed in the fetus while nevertheless being constantly transcribed in the extra-embryonic tissues due to the lower DNA methylation level in these tissues (Kaneko-Ishino and Ishino, 2012, 2015). Therefore, ERV-derived genes might have been functionally selected under specific circumstances in the extra-embryonic tissues (Kaneko-Ishino and Ishino, 2015) (Fig. 11). This would suggest the extra-embryonic tissues serve as a cradle or incubator for retrovirus-derived genes. This may be consistent with and/or complementary to the recently reported finding of the placenta serving as a dumping ground for genetic defects (Coorens et al., 2021), because the placenta is thus able to tolerate major genetic and/or developmental flaws, a capacity that affords a tremendous advantage for the survival of the fetus. Our work indicates a previously unreported role for the yolk sac in the functional evolution of the innate immune system in eutherians.

## MATERIALS AND METHODS

### Mice

All of the animal experiments were reviewed and approved by Institutional Animal Care and Use Committee of RIKEN Kobe Branch, Osaka University, Tokai University and Tokyo Medical and Dental University (TMDU), and were performed in accordance with the RIKEN Guiding Principles for the Care and Use of Laboratory Animals, as well as the Guideline for the Care and Use of Laboratory Animals of Osaka University, Tokai University and TMDU.

### Comparative genome analysis

The sushi-ichi GAG (AAC33525.1), mouse RTL5 (NP_001265463.1) and RTL6 (NP_808298.2) protein sequences were obtained from NCBI and Ensemble. Amino acid identity and similarity were calculated using the EMBOSS Water program (http://www.ebi.ac.uk/Tools/psa/emboss_water/) in the default mode. The orthologues of *RTL5* and *RTL6* were identified by a search of the NCBI Gene database (http://www.ncbi.nlm.nih.gov/gene/) using *RTL5* (and *RGAG4*) and *RTL6* (and *LDOC1L*) as the query terms. Genomic homology analysis was performed using the mVISTA LAGAN program (http://genome.lbl.gov/vista/mvista/submit.shtml).

The sequences used for *RTL5* comparative genome analysis in Fig. 1A were as follows: chicken (*Gallus gallus*), NC_052535.1[c2173485-1600210]; platypus (*Ornithorhynchus anatinus*), NC_041733.1[17047115-18009974]; echidna (*Tachyglossus aculeatus*), NC_052071.1[c42206678-41135833]; opossum (*Monodelphis domestica*), NC_008809.1[c72655043-72430642]; mouse (*Mus musculus*), NC_000086.8[100676255-101558086]; human (*Homo sapiens*), NC_000023.11[71526681-72577989]; chimpanzee (*Pan troglodytes*), NC_036902.1[66945448-68002204]; cattle (*Bos taurus*), NC_037357.1[c79138511-78100425]; dog (*Canis lupus familiaris*), NC_051843.1[56788745-57634597]; cat (*Felis catus*), NC_018741.3[60765912-61585357]; horse (*Equus caballus*), NC_009175.3[56412010-57205718]; and African savanna elephant (*Loxodonta africana*), NW_003573444.1[c31006766-29638658].

For *RTL6*, we obtained the *SHISAL1*-*PRR5* genomic sequences in Fig. 1B from the NCBI database. The sequences used for analysis were as follows: chicken (*Gallus gallus*), NC_006088.4[69115783-70071731]; platypus (*Ornithorhynchus anatinus*), NC_041741.1[c52020074-51756772]; echidna (*Tachyglossus aculeatus*), NC_052079.1[c58674342-58403296]; opossum (*Monodelphis domestica*), NC_008808.1[15497440-16650585] and NW_001583545.1[1-415929]; Tasmanian devil (*Sarcophilus harrisii*), NW_003843556.1[1-720570] and NW_003844018.1[1-136229]; mouse (*Mus musculus*), NC_000081.6[84153625-84874824]; human (*Homo sapiens*), NC_000022.11[43913843-45028826]; dog (*Canis lupus familiaris*), NC_006592.3[21015698-21743182]; African savanna elephant (*Loxodonta africana*), NW_003573493.1[76766-1008174]; armadillo (*Dasypus novemcinctus*), NW_004489016.1[1-309452]; and sloth (*Choloepus hoffmanni*), KN190031.1[1-672150].

### Estimation of the pairwise dN/dS ratio

The nonsynonymous/synonymous substitution rate ratio (dN/dS) was estimated with CodeML (runmode: −2) in PAML (Xu and Yang, 2013). An amino acid sequence phylogenic tree was constructed with MEGA7 (Kumar et al., 2016) using the Maximum Likelihood method based on the JTT matrix-based model. The codon alignment of cDNA was created with the PAL2NAL program (www.bork.embl.de/pal2nal/) (Suyama et al., 2006).

The *RTL5* genome sequences used for the dN/dS analysis (Table 1) were the following: mouse, NC_000086.8[c101114468-101112669]; human, NC_000023.11[c72131540-72129831]; chimpanzee, NC_036902.1[c67554437-67552728]; dog, NC_051843.1[c57238370-57236637]; cattle, NC_037357.1[78545112-78546836]; horse, NC_009175.3[c56844294-56842597]; elephant, NW_003573444.1[30233756-30235345]; manatee, NW_004444006.1[c9027102-9025528]; armadillo, NW_004489802.1[c991100-989476]; sloth, KN181417.1[c16728-15101].

The *RTL6* genome sequences used for analysis were the following: mouse, NC_000081.6[84556462-84557193]; human, NC_000022.11[44496837-44497556]; chimpanzee, NC_006489.4[31270844-31271566]; dog, NC_006592.3[21316309-21317028]; cattle, AC_000162.1[115850420-115851139]; horse, NC_009171.2[40642984-40643703]; elephant, NW_003573493.1[337200-337919]; manatee, NW_004444005.1[6540826-6541545]; armadillo, NW_004489016.1[146135-146854]; sloth, KN190031.1[86985-87704].

### Rapid amplification of cDNA Ends (RACE)

For the 5′-RACE experiment, the 5′-Full RACE Core Set (TaKaRa) was used to extend *Rtl6* mRNA from the mouse brain at 8 weeks of age, according to the manufacturer's instructions. The first-strand cDNA synthesis was carried out with 1 μg of total RNA using Rtl6 5′-RACE GSP (Fig. 2A): 5′-AGGGTGTCAACTACG-3′ (5′-phospholylated). The 5′-RACE PCR was performed with Ex*Taq* polymerase (TaKaRa) using the following primers: Rtl6-race-F1, 5′-CTGAAAGCCCAGCCTCTGCC-3′; and Rtl6-race-R1, 5′-TGGAGGTCCGAGGTTGGACC-3′. The 5′-RACE PCR products (1st 5′-RACE products) were confirmed by 1.5% agarose gel electrophoresis. Nested PCR was performed with Ex*Taq* polymerase using the following primers: Rtl6-race-F2, 5′-TGGTGCCAGCGCTCAGATGG-3′ and Rtl6-race-R2, 5′-AGGTTGGACCATGCTGGCGG-3′. A 1/50 dilution of the 1st 5′-RACE products was used as a template. The nested PCR products were separated by 1.5% agarose gel electrophoresis and extracted from the gel. The extracted PCR products were cloned into a pGEM-Teasy vector (Promega). The DNA sequence was determined by Sanger sequencing. For the 3′-RACE experiment, the 3′-Full RACE Core Set (TaKaRa) was used to extend *Rtl6* mRNA from the mouse brain at 8 weeks of age according to the manufacturer's instructions. First-strand cDNA synthesis was carried out with 1 μg of total RNA. The 3′-RACE PCR was performed with Ex*Taq* polymerase (TaKaRa) using the following primers: Rtl6 3′-RACE GSP, 5′-ATCCAGCCTCCAACGGGACC-3′; and three sites adaptor primer, 5′-CTGATCTAGAGGTACCGGATCC-3′. The 3′-RACE PCR products (1st 3′-RACE products) were confirmed by 1% agarose gel electrophoresis. Semi-nested PCR was performed with Ex*Taq* polymerase using the following primers: Rtl6-race-F4, 5′-TCCAACGGGACCAATCCCGC-3′**;** and three sites of adaptor primer (see above). A 1/50 dilution of the first 3′-RACE product was used as a template. The semi-nested PCR products were separated by 1% agarose gel electrophoresis and extracted from the gel. The extracted PCR products were cloned into a pGEM-Teasy vector (Promega). The DNA sequence was determined by Sanger sequencing.

### Generation of the *Rtl6-Venus* knock-in mice

The *Rtl6-Venus* fusion protein construct (pRtl6CV) was generated using Gateway cloning technology (Thermo Fisher Scientific) and the method of Kuroyanagi et al. (2010). The PCR fragment, including the Rtl6 5′-UTR to ORF (the Rtl6N fragment), was generated using PrimeSTAR Max DNA Polymerase (TaKaRa) and the following primers: Rtl6attB1, 5′-GGGGACAAGTTTGTACAAAAAAGCAGGCTCAACCGAAGGATGAGAGGGTC-3′; and Rtl6NattB5r, 5′-GGGGACAACTTTTGTATACAAAGTTGTCCGAGGTTGGACCATGCTGGCG-3′. The PCR fragment, including the Rtl6 5′-UTR to Sirh3 ORF end (the Rtl6C fragment), was generated using PrimeSTAR Max DNA Polymerase (TaKaRa) and the following primers: Rtl6attB1 and Rtl6CattB5r, 5′-GGGGACAACTTTTGTATACAAAGTTGTAAGGTTCCGGCCACGAGAGGGCA-3′.

The pDONRRtl6N and pDONRRtl6C vectors were constructed by the Gateway BP reaction using the following fragments and vectors: pDONRRtl6N, the Rtl6N fragment and pDONR221 P1-P5r (Thermo Fisher); pDONRRtl6C, the Rtl6C fragment and pDONR221 P1-P5r. The Rtl6-3′ UTR fragment was obtained by PCR amplification using the following primers: Rtl6XhoI-F, 5′-CGCCTCGAGGGACTTGCCACCACCCTGGTAG-3′; and Rtl6EcoRI-R, 5′-CGCGAATTCCTCCTGTCCTGGTCTTGCAAAGG-3′. The Rtl6-3′ UTR fragment was ligated to the XhoI- and EcoRI-digested pBluescript SK(+) vector. The inverse-PCR fragment of Rtl6-3′ UTR was amplified using the following primers: Rtl6attB1, 5′-GGGGAGCCTGCTTTTTTGTACAAACTTGTCCGGTACCCAATTCGCCCTATAG-3′; and Rtl6attB2, 5′-GGGGACCCAGCTTTCTTGTACAAAGTGGTCGGACTTGCCACCACCCTGGTAG-3′. The pDONRRtl6-3′ UTR vector was constructed by the Gateway BP reaction using pDONR221 P1-P2 (Thermofisher) and the Rtl6-3′ UTR fragment. The pRtl6NV and pRtl6CV vectors were constructed by the Gateway LP reaction using the following vectors: pRtl6NV (pDONRRtl6N, pDONRRtl6-3′ UTR and pENTR-L5-Venus-L2); pRtl6CV (pDONRRtl6C, pDONRRtl6-3′UTR and pENTR-L5-Venus-L2).

To generate the *Rtl6* KI targeting vector, we obtained two PCR fragments, the 5′-arm (6.5 kb) and 3′-arm (3.5 kb), using PrimeSTAR Max DNA Polymerase (TaKaRa). The C57BL/6N genome was used as the PCR template. For Rtl6 5′- and 3′-arm cloning, we used the following primers: 5′-arm Rtl6KI-LA-F1, 5′- CTGACTgtcgaccaattgCCTGCTGTTTGGTGGTTGAGCCTCTG-3′; 5′-arm Rtl6KI-LA-R1, 5′-CTGACTggattcCTTTACGATTCCTACCCAGGCCGCTC-3′; 5′-arm Rtl6KI-LA-F2, 5′-CTGACTgtcgacGGGAATGTAGAGGCAGGAGAGGTTCAAGG-3′; 5′-arm Rtl6KI-LA-R2, 5′-CTGACTgcggccgcttaattaaGGAGTGTTCCAGGAGCTGAGTATCCGTG-3′; 3′-arm Rtl6KI-SA-F1, 5′-CTGACTgtcgacCTACAGCTCTTGCTGCCCCAGGC-3′; 3′-arm Rtl6KI-SA-R1, 5′-CTGACTgcggccgcGTGTGGGCTGAAGACAGGTGGGTTG-3′. The middle arm (1 kb, Rtl6NV; 1.5 kb, Rtl6CV) fragments were generated by restriction enzyme digestion of pRtl6NV and pRtl6CV, respectively. All of the arm fragments were inserted into a pNT1.1 vector.

The establishment of knock-in ES cells and generation of chimeric mice were conducted as previously described (Fujihara et al., 2013). In brief, EGR-G101 ES cells were electroporated with linearized DNA, and then screened by PCR after positive/negative selection. Chimeric mice were produced by the eight-cell microinjection method. To remove the flox region, we injected a pCAG/NCre plasmid (Sato et al., 2000) into the fertilized eggs generated by *in vitro* fertilization (IVF) from C57BL/6N eggs and Rtl6-CV mutant sperm.

### Immunoprecipitation and western blotting

Adult brain (8 weeks) was dissected into seven parts and each part was powderized in liquid N_2_ using a Multi-beads shocker (MB1050, Yasui Kikai). The powder samples of wild-type and RTL6-CV cerebrum (46 mg and 48.1 mg, respectively) were dissolved in 150 µl of RIPA buffer, 50 mM Tris-HCl (pH 8.0), 150 mM NaCl, 0.5% sodium deoxycholate, 0.1% SDS, 1% NP-40 (IGEPAL CA-630) and 1 mM EDTA supplemented with 20× protease inhibitor solution (Sigma-Aldrich, P2714) on ice for 30 min. After 20 min of centrifugation (10,000 ***g***, at 4°C), the supernatant was mixed with anti-GFP (RatIgG2a), monoclonal (GF090R), CC, agarose conjugate (Nacalai Tesque) and incubated overnight at 4°C. The agarose beads were washed four times with 500 µl of RinseBuffer, 50 mM Tris-HCl (pH 8.0) and 150 mM NaCl at 4°C. Then the beads were incubated with 60 µl of SDS sample buffer and directly applied to gel electrophoresis using a 10% acrylamide gel. Western blot analysis was performed using a standard protocol. After blotting on a Hybond-P (GE Healthcare) membrane, the Sirh3-Venus fusion protein was detected with an ECL Prime Western Blotting Detection kit (GE Healthcare) using an anti-GFP antibody (MBL, 598) and an anti-rabbit goat immuno-globulins/HRP (DAKO, P0160) as the 1st and 2nd antibodies. Signals were detected with an AE-9300 Ez CaptureMG (Atto).

### Quantitative RT-PCR

Total RNA was prepared from frozen tissues using ISOGEN (Nippon Gene) and ISOGEN-LS (Nippon Gene). The cDNA was made from total RNA (1 μg) using Revertra Ace qPCR RT Master Mix (Toyobo). Quantitative RT-PCR analysis was performed using Fast SYBR Green Master Mix (Life Technologies) and a StepOnePlus System (ABI) by means of an absolute quantification method. An unpaired Student's *t*-test was used for statistical analysis. The following primer sequences were used: Actb-F, 5′-AAGTGTGACGTTGACATCCG-3′; Actb-R, 5′-GATCCACATCTGCTGGAAGG-3′; Rtl6-F2, 5′-GTGTTGGGTGGCAAATGCTCGG-3′; Rtl6-R2, 5′-GGACCTCCCAGACACTGCAAGC-3′.

### Imaging using confocal laser scanning fluorescence microscope

Fresh brain and brain slices (2 mm in depth) from *Rtl5*-CmCherry and *Rtl6*-CVenus KI mice were used for analysis with a ZEISS LSM880 (Zeiss) with and without fixation using 4% paraformaldehyde (PFA). Samples were covered with 10% glycerol solution for protection from drying. The samples were observed using a Plan-Apochromat lens (10×, numerical aperture 0.45, M27, Zeiss) and a C-Apochromat lens (63× numerical aperture 1.2 water, Zeiss). The tiling with lambda-mode images was obtained using the following settings: pixel dwell, 1.54 μs; average, line 4; master gain, 1250 for ChS and 542 for ChD; pinhole size, 33 µm; filter, 500-696 nm; beam splitter, MBS 458/514; lasers, 514 nm (Argon 514) 0.90%. For the tiling-scan observations, the images were captured as 84 tiles as follows: overlap in percentage, 10.0; tiling mode, rectangular grid; size, *x*=15,442.39 µm and *y*=9065.95 µm. Spectral unmixing and processing of the obtained images were conducted using ZEN imaging software (Zeiss). The spectrum from the Venus proteins (Maximum peak emission fluorescence wavelength: 528 nm) was detected only in the samples from *Rtl6*-CV, and from *Rtl5-CmC* and *Rtl6*-CV double knock-in mice, not the wild-type control samples. The mCherry signal (maximum peak emission fluorescence wavelength 610 nm) detected in *Rtl5-CmC*, and in *Rtl5-CmC* and *Rtl6*-CV double knock-in mice was distinguished from Af610 nm using the peak shape, e.g. the width and/or co-existence of a second peak. The relative intensity of RTL5-mCherry (red), RTL6-Venus (green) and LPS (blue) signals along the *x*-axis of the brain (from the olfactory bulb to the cerebellum regions) was calculated from the 3D scanning data. The total intensity of each signal on and above each *y*-axis was summed, divided by the transmission signal and presented as the relative signal intensity on the *y*-axis in this figure.

### Immunostaining

For immunostaining, fresh frozen optimal cutting temperature (OCT) compound (Tissue-Tek, Sakura, FineTek)-embedded brain sections were fixed in 4% PFA (26126-25, Nacalai Tesque) in 0.1 M PBS at room temperature for 20 min and washed three times with PBS for 5 min. Then they were treated with PBS containing 0.2% Triton-X100 for 20 min and washed three times with PBS for 5 min. They were next stained with DAPI (250 ng/ml) for 10 min at room temperature and washed three times with PBS for 5 min. After carrying out the blocking reaction using PBS containing 5% normal goat serum and 1% BSA at room temperature for 30 min, the sections were first incubated with an anti-Iba1 rabbit antibody (MBL, D513-A48, 1/1000 dilution) or anti-TMEM119 rabbit antibody (Abcam, ab209064, 1/1000 dilution). After being washed three times with PBS for 5 min, they were incubated with an anti-rabbit-Alexa Fluor 488 antibody (MBL, D533-A62, 1/1000 dilution) at 4°C overnight and finally washed three times with PBS for 5 min, and the images were captured with a LSM880 (Zeiss).

### Generation of the *Rtl5-mCherry* knock-in mice

*Rtl5-CmCherry* mouse was generated by pronuclear microinjection of the CRISPR/Cas system (see Fig. 5A), essentially as in a previous report (Aida et al., 2015), using single-stranded (ss) donor DNA. ssDNA was prepared by 5′phosphorylated primer-mediated PCR from the template plasmid followed by digestion of the phosphorylated strand with lambda exonuclease. The template plasmid for the mCherry-targeting ssDNA was constructed with 1.5 kb long 5′ and 3′ arms amplified from the C57BL/6N genome using PrimeSTAR GXL DNA Polymerase (TaKaRa), the upstream genome sequence of the stop codon of Rtl5 and downstream of the predictive cut site by Cas9, respectively. The C-terminus of Rtl5 was fused with mCherry by means of a cloning enzyme (In-Fusion HD Cloning Kit, Takara Bio) and it was inserted into a pGEM T Easy vector (Promega). A T to A silent mutation was introduced into the position of *Rtl5* Threonine 593 (ACT to ACA) to inhibit recutting of the genomic DNA after editing with the CRISPR/Cas system.

Double-stranded DNA (dsDNA) fragments (1142 base pairs) containing 5′ and 3′ homology arms (233 and 201 base pairs, respectively) and an mCherry-coding sequence were amplified from the template plasmid by PCR using PrimeSTAR GLX DNA polymerase (TAKARA) along with the sense and antisense primer pair (5′-ATTACCTGGGGGTATCCCCTT-3′ and 5′-CACTCTTCTGGTTGTGGTTGC-3′). The antisense primer was pre-phosphorylated at its 5′ end (Fasmac). Amplified dsDNA fragments were column purified using a MiniElute PCR Purification Kit (QIAGEN) according to the manufacturer's recommendations.

Ten μg of the purified dsDNA were treated with Lambda exonuclease (NEB) in 50 μl of reaction solution at 37°C for 30 min and then at 75°C for 10 min to digest the phosphorylated strand and produce single-stranded DNA (ssDNA). Part of the reaction solution was analyzed for digestion efficiency and ssDNA production by electrophoresis using 1% agarose gel. Total ssDNA was gel electrophoresed following exonuclease treatment and quality assessment, and gel purified using a Long ssDNA Preparation Kit for 3 kb (Biodynamics Laboratory) according to the manufacturer's recommendations (∼2 μg of ssDNA was produced from 10 μg of amplified dsDNA). Purified ssDNA was stored at 4°C until required, assessed for its quality by Sanger sequencing and injected into mouse pronuclei at a final concentration of 5 ng/μl within 1 week of preparation. Immediately before pronuclear injection, ssDNA was mixed with other components of the CRISPR/Cas system (Cas9 protein, crRNA and tracrRNA).

### LPS, dsRNA and non-methylated DNA injection into the brain

*Rtl5*-CmC and *Rtl6*-CV double hetero mice (P2 neonates to 5w young adults) were used for the injection experiments after being anesthetized with isoflurane. Approximately 10-20 µl of Alexa 488-labeled LPS (Invitrogen, L23351), Alexa 594-labeled LPS (Invitrogen, L23353), rhodamine-labeled dsRNA (MBL, Code No. D488-A24) and cyanogen 3-labeled nonmethylated dsDNA (see below) were injected using 20 ng/µl solution, respectively, and 1 ml insulin syringes and a 26 G needle. 1 min after the injection, the needle was pulled out and kept out for ∼5-10 min, then the fresh brain was dissected out in ice-cold PBS solution. The inner surface of the brain hemispheres was analyzed with a Zeiss LSM880 before and after fixation with 4% PFA. Cyanogen 3-labeled nonmethylated dsDNA was made by mixing two complementary oligo DNAs, heating at 60°C for 5 min and annealing at room temperature. The oligo DNAs used were 5′-cyanogen 3-labelled GACGTTGACGTTGACGTTGACGTT and 5′-cyanogen 3-labeled AACGTCAACGTCAACGTCAACGTC.

### Generation of *Rtl6* KO mice

To generate *Rtl6* MT mice (CDB0556K: http://www.clst.riken.jp/arg/mutant%20mice%20list.html), we obtained three genomic fragments, the 5′-arm (3.5 kb, NC_000081.6[84557206-84560780]), middle arm (0.8 kb, NC_000081.6[84556384-84557205]) and 3′-arm (6.8 kb, NC_000081.6[84549544-84556383]) by recombination from the R23-74I9 BAC clone (BACPAC Resources), and then cloned them into a targeting vector. The targeting vector was introduced by electroporation into TT2 ES cells (C57BL/6×CBA genetic background) (Yagi et al., 1993). ES cells in which homologous recombination had occurred were injected into eight-cell stage embryos. Germline transmission of the *Rtl6* MT allele was confirmed by Southern blot and PCR using the genome prepared from pups in which male *Rtl6* chimeric mice had been crossed with female C57BL/6J. To remove the flox region, we injected a pCAG/NCre plasmid (Sato et al., 2000) into the fertilized eggs that had been generated by *in vitro* fertilization (IVF) from C57BL/6J eggs and *Rtl6* MT hetero sperm, thus establishing *Rtl6* neo mice. To obtain *Rtl6* KO mice, we injected a pCAGGS-FLPe plasmid (Gene bridge) into the fertilized eggs generated by IVF from the C57BL/6J eggs and *Rtl6* neo hetero sperm. Exclusion of the neo cassette was confirmed by genomic PCR of the pups' DNA. Southern blot analysis was performed using a standard protocol. Genomic DNA (5 μg) from the tail were digested by the restriction enzyme SpeI (TaKaRa). Hybond-N+ (GE Healthcare) membranes blotted with digested DNA were hybridized in Church buffer with radio isotope-labelled probes. The 5′ and 3′ probes were generated by genomic PCR using the following sequences: 5′ probe, NC_000081.6[84561538-84561968]; and 3′ probe, NC_000081.6[84545196-84545715]. The *Rtl6* KO allele was detected by genomic PCR. Genomic DNA was prepared from the tail or ear tip using a DNeasy Blood & Tissue Kit (Qiagen). Genotyping PCR was performed using Ex*Taq*HS polymerase (TaKaRa) with the following primers (wild type, 1277 bp; knockout, 707 bp): Rtl6-F1, 5′-TATCCAGCTCGAGCATCCTT-3′; and Rtl6-R4, 5′-CAGCAAGAGCTGTAGGGAGTGTT-3′. *Rtl6* KO mice were backcrossed to C57BL6/J for more than 10 generations.

### Generation of the *Rtl5* KO mice

To generate the *Rtl5* KO targeting vector, we obtained four PCR fragments, the long arm 1 (2.7 kb), long arm 2 (3.9 kb), middle arm (2.9 kb) and short arm (3.4 kb), using PrimeSTAR Max DNA Polymerase (TaKaRa). The C57BL/6N genome was used as the PCR template. For *Rtl5* long arm cloning, we used the following primers: Rtl5KO-LA-F1, 5′-GGAAGTTGGGTTCTTGCCCATCACC-3′; Rtl5KO-LA-R1, 5′-TCATCAAAGGCAGCTGGAGCTTGAC-3′; Rtl5KO-LA-F2, 5′-AGCCAGCTTAACTGTGGATGAGAC-3′; and Rtl5KO-LA-R2+SalI, 5′-ACGCgtcgacTGTGACAAGTTCCTGGGGCTTTGAG-3′.

For *Rtl5* middle arm cloning, we used the following primers: Rtl5KO-MA-F1+BamHI, 5′-CGCggatccAGGCATGGGCTATATAACAGGAGGG-3′; and Rtl5KO-MA-R1+SalI, 5′-ACGCgtcgacGGAATTCTGCAGGTCTTCTTTGGG-3′. For *Rtl5* short arm cloning, we used the following primers: Rtl5KO-SA-F1+BamHI, 5′-CGCggatccACACTGGGGGTGAGTTGGCCACG-3′; and Rtl5KO-SA-R1+SalI, 5′-ACGCgtcgacGGACAATCAGCCACACGCTCAGCAC-3′. Each of four fragments was subcloned into the pUC19 vector and, respectively, called LA1, LA2, MA and SA vector. The long arm vector (LA vector) was generated by insertion of the LA1 fragment digested by KpnI and BamHI restriction enzyme into the LA2 vector. To insert two loxP sites inside the RTL5 ORF, two inverse PCRs were performed on the MA vector using the following primers: Rtl5KO-5′loxPc, 5′-ATAACTTCGTATAATGTATGCTATACGAAGTTATTAgtgcctttcctgtgctggcc-3′; Rtl5KO-3′loxP+stop+AscI-F, 5′-ataacttcgtatagcatacattatacgaagttatTATGATGATAATAGggcgcgccACACTGG-3′; Rtl5KO-5′loxP, 5′-ATAACTTCGTATAGCATACATTATACGAAGTTATTAgtgcctttcctgtgctggcc-3′; and Rtl5KO-3′loxpC+stop+AscI-F, 5′-ataacttcgtataatgtatgctatacgaagttatTATGATGATAATAGggcgcgccACACTGGGGGTGAGTTGGCC-3′. All of the three arm fragments, LA, MA and SA, were inserted into a pNT1.1 vector.

To generate chimeric mice, we introduced the *Rtl5* KO targeting vector into the ES cells [EGR-G101, cag/acr-EGFP(B6N)]. The homologous recombination in the ES cells was confirmed by genomic PCR and Southern blotting. Germline transmission of *Rtl5* mutant allele was confirmed by PCR using the genome prepared from the pups in which male *Rtl5* chimeric mice had been crossed with female C57BL/6N. To remove the flox region, we injected a pCAG/NCre plasmid into the fertilized eggs generated by *in vitro* fertilization (IVF) from C57BL/6N eggs and *Rtl5* mutant sperm. To remove the neomycin cassette, we injected a pCAGGS-FLPe plasmid (Gene bridge) into the fertilized eggs generated by IVF from the C57BL6/N eggs and *Rtl5* KO sperm that still had the neo cassette.

## Supplementary Material

10.1242/develop.200976_sup1 Supplementary information

## References

[DEV200976C1] Aida, T., Chiyo, K., Usami, T., Ishikubo, H., Imahashi, R., Wada, Y., Tanaka, K. F., Sakuma, T., Yamamoto, T. and Tanaka, K. (2015). Cloning-free CRISPR/Cas system facilitates functional cassette knock-in in mice. *Genome Biol.* 16, 87. 10.1186/s13059-015-0653-x 25924609 PMC4414275

[DEV200976C2] Ashley, J., Cordy, B., Lucia, D., Fradkin, L. G., Budnik, V. and Thomson, T. (2018). Retrovirus-like Gag protein Arc1 binds RNA and traffics across synaptic boutons. *Cell* 172, 262-274.e11. 10.1016/j.cell.2017.12.022 29328915 PMC5793882

[DEV200976C3] Bennett, M. L., Bennett, F. C., Liddelow, S. A., Ajami, B., Zamanian, J. L., Fernhoff, N. B., Mulinyawe, S. B., Bohlen, C. J., Adil, A., Tucker, A. et al. (2016). New tools for studying microglia in the mouse and human CNS. *Proc. Natl. Acad. Sci. USA* 113, E1738-E1746. 10.1073/pnas.1525528113 26884166 PMC4812770

[DEV200976C5] Brandt, J., Schrauth, S., Veith, A.-M., Froschauer, A., Haneke, T., Schultheis, C., Gessler, M., Leimeister, C. and Volff, J.-N. (2005). Transposable elements as a source of genetic innovation: expression and evolution of a family of retrotransposon-derived neogenes in mammals. *Gene* 345, 101-111. 10.1016/j.gene.2004.11.022 15716091

[DEV200976C6] Brosius, J. and Gould, S. J. (1992). On “genomenclature”: a comprehensive (and respectful) taxonomy for pseudogenes and other “junk DNA”. *Proc. Natl. Acad. Sci. USA* 89, 10706-10710. 10.1073/pnas.89.22.10706 1279691 PMC50410

[DEV200976C7] Calvo, S. E., Pagliarini, D. J. and Mootha, V. K. (2009). Upstream open reading frames cause widespread reduction of protein expression and are polymorphic among humans. *Proc. Natl. Acad. Sci. USA* 106, 7507-7512. 10.1073/pnas.0810916106 19372376 PMC2669787

[DEV200976C8] Chikamori, H., Ishida, Y., Nakamura, Y., Koyama, Y. and Yamada, S. (2019). Distinctive expression pattern of Peg10 in the mouse brain. *Eur. J. Anat.* 23, 361-368.

[DEV200976C9] Coorens, T. H. H., Oliver, T. R. W., Sanghvi, R., Sovio, U., Cook, E., Vento-Tormo, R., Haniffa, M., Young, M. D., Rahbari, R., Sebire, N. et al. (2021). Inherent mosaicism and extensive mutation of human placentas. *Nature* 592, 80-85. 10.1038/s41586-021-03345-1 33692543 PMC7611644

[DEV200976C10] Fiebich, B. L., Batista, C. R. A., Saliba, S. W., Yousif, N. M. and de Oliveira, A. C. P. (2018). Role of microglia TLRs in neurodegeneration. *Front. Cell. Neurosci.* 12, 329. 10.3389/fncel.2018.00329 30333729 PMC6176466

[DEV200976C11] Floden, A. M. and Combs, C. K. (2007). Microglia repetitively isolated from in vitro mixed glial cultures retain their initial phenotype. *J. Neurosci. Methods.* 164, 218-224. 10.1016/j.jneumeth.2007.04.018 17553568 PMC2041803

[DEV200976C12] Fujihara, Y., Kaseda, K., Inoue, N., Ikawa, M. and Okabe, M. (2013). Production of mouse pups from germline transmission-failed knockout chimeras. *Transgenic Res.* 22, 195-200. 10.1007/s11248-012-9635-x 22826106

[DEV200976C13] Ginhoux, F., Greter, M., Leboeuf, M., Nandi, S., See, P., Gokhan, S., Mehler, M. F., Conway, S. J., Ng, L. G., Stanley, E. R. et al. (2010). Fate mapping analysis reveals that adult migroglia derive from primitive macrophages. *Science* 330, 841-845. 10.1126/science.1194637 20966214 PMC3719181

[DEV200976C14] Ginhoux, F., Lim, S., Hoeffel, G., Low, D. and Huber, T. (2013). Origin and differentiation of microglia. *Front. Cell. Neurosci.* 7, 45. 10.3389/fncel.2013.00045 23616747 PMC3627983

[DEV200976C15] Gould, S. J. and Vrba, E. S. (1982). Exaptation – a missing term in the science of form. *Paleobiology* 8, 4-15. 10.1017/S0094837300004310

[DEV200976C16] Hanisch, U.-K. and Kettenmann, H. (2007). Microglia: active sensor and versatile effector cells in the normal and pathologic brain. *Nat. Neurosci.* 10, 1387-1394. 10.1038/nn1997 17965659

[DEV200976C17] Hinnebusch, A. G., Ivanov, I. P. and Sonenberg, N. (2016). Translational control by 5′-untranslated regions of eukaryotic mRNAs. *Science* 352, 1413-1416. 10.1126/science.aad9868 27313038 PMC7422601

[DEV200976C18] Hornik, T. C., Neniskyte, U. and Brown, G. C. (2014). Inflammation induces multinucleation of microglia via PKC inhibition of cytokinesis, generating highly phagocytic multinucleated giant cells. *J. Neurochem.* 128, 650-661. 10.1111/jnc.12477 24117490

[DEV200976C19] Irie, M., Yoshikawa, M., Ono, R., Iwafune, H., Furuse, T., Yamada, I., Wakana, S., Yamashita, Y., Abe, T., Ishino, F. et al. (2015). Cognitive function related to the *Sirh11/Zcchc16* gene acquired from an LTR retrotransposon in eutherians. *PLoS Genet.* 11, e1005521. 10.1371/journal.pgen.1005521 26402067 PMC4581854

[DEV200976C20] Ito, D., Imai, Y., Ohsawa, K., Nakajima, K., Fukuuchi, Y. and Kohsaka, S. (1998). Microglia-specific localisation of a novel calcium binging protein, Iba1. *Brain Res. Mol. Brain Res.* 57, 1-9. 10.1016/S0169-328X(98)00040-0 9630473

[DEV200976C21] Jumper, J., Evans, R., Pritzel, A., Green, T., Figurnov, M., Ronneberger, O., Tunyasuvunakool, K., Bates, R., Žídek, A., Potapenko, A. et al. (2021). Highly accurate protein structure prediction with AlphaFold. *Nature* 596, 583-589. 10.1038/s41586-021-03819-2 34265844 PMC8371605

[DEV200976C22] Kagami, M., Sekita, Y., Nishimura, G., Irie, M., Kato, F., Okada, M., Yamamori, S., Kishimoto, H., Nakayama, M., Tanaka, Y. et al. (2008). Deletions and epimutations affecting the human 14q32.2 imprinted region in individuals with paternal and maternal upd(14)-like phenotypes. *Nat. Genet.* 40, 237-242. 10.1038/ng.2007.56 18176563

[DEV200976C23] Kaneko-Ishino, T. and Ishino, F. (2012). The role of genes domesticated from LTR retrotransposons and retroviruses in mammals. *Front. Microbiol.* 3, 262. 10.3389/fmicb.2012.00262 22866050 PMC3406341

[DEV200976C24] Kaneko-Ishino, T. and Ishino, F. (2015). Mammalian-specific genomic functions: Newly acquired traits generated by genomic imprinting and LTR retrotransposon-derived genes in mammals. *Proc. Jpn. Acad. Ser. B Phys. Biol. Sci.* 91, 511-538. 10.2183/pjab.91.511 PMC4773580 26666304

[DEV200976C25] Kayagaki, N., Wong, M. T., Stowe, I. B., Ramani, S. R., Gonzalez, L. C., Akashi-Takamura, S., Miyake, K., Zhang, J., Lee, W. P., Muszyński, A. et al. (2013). Noncanonical inflammasome activation by intracellular LPS independent of TLR4. *Science* 341, 1246-1249. 10.1126/science.1240248 23887873

[DEV200976C26] Kim, A., Terzian, C., Santamaria, P., Pélisson, A., Purd'homme, N. and Bucheton, A. (1994). Retroviruses in invertebrates: The gypsy retrotransposon is apparently an infectious retrovirus of Drosophila melanogaster. *Proc. Natl. Acad. Sci. USA* 91, 1285-1289. 10.1073/pnas.91.4.1285 8108403 PMC43142

[DEV200976C27] Kimura, M. (1986). DNA and the neutral theory. *Philos. Trans. R Soc. Lond. B Biol. Sci.* 312, 343-354. 10.1098/rstb.1986.0012. 2870526

[DEV200976C28] Kitazawa, M., Hayashi, S., Imamura, M., Takeda, S., Oishi, Y., Kaneko-Ishino, T. and Ishino, F. (2020). Deficiency and overexpression of *Rtl1* in the mouse cause distinct muscle abnormalities related to Temple and Kagami-Ogata syndromes. *Development* 147, dev185918. 10.1242/dev.185918 32878913 PMC7490516

[DEV200976C29] Kitazawa, M., Sutani, A., Kaneko-Ishino, T. and Ishino, F. (2021). The role of eutherian-specific *RTL1* in the nervous system and its implications for the Kagami-Ogata and Temple syndromes. *Genes Cells* 26, 165-179. 10.1111/gtc.12830 33484574 PMC7986171

[DEV200976C30] Kumar, S., Stecher, G. and Tamura, K. (2016). MEGA7: Molecular Evolutionary Genetics Analysis Version 7.0 for Bigger Datasets. *Mol. Biol. Evol.* 33, 1870-1874. 10.1093/molbev/msw054 27004904 PMC8210823

[DEV200976C31] Kuroyanagi, H., Ohno, G., Sakane, H., Maruoka, H. and Hagiwara, M. (2010). Visualization and genetic analysis of alternative splicing regulation in vivo using fluorescence reporters in transgenic Caenorhabditis elegans. *Nat. Protoc.* 5, 1495-1517. 10.1038/nprot.2010.107 20725066

[DEV200976C32] Lian, H., Roy, E. and Zheng, H. (2016). Protocol for primary microglial culture preparation. *Bio. Protoc.* 6, e1989. 10.21769/BioProtoc.1989 PMC5669279 29104890

[DEV200976C33] Mazgaeen, L. and Gurung, P. (2020). Recent advances in lipopolysaccharide recognition systems. *Int. J. Mol. Sci.* 21, 379. 10.3390/ijms21020379 PMC7013859 31936182

[DEV200976C34] Meseguer, V., Alpizar, Y. A., Luis, E., Tajada, S., Denlinger, B., Fajardo, O., Manenschijn, J.-A., Fernández-Peña, C., Talavera, A., Kichko, T. et al. (2014). TRPA1 channels mediate acute neurogenic inflammation and pain produced by bacterial endotoxins. *Nat. Commun.* 5, 3125. 10.1038/ncomms4125 24445575 PMC3905718

[DEV200976C35] Mirdita, M., Schütze, K., Moriwaki, Y., Heo, L., Ovchinnikov, S. and Steinegger, M. (2022). ColabFold: Making protein folding accessible to all. *Nat. Methods* 19, 679-682. 10.1038/s41592-022-01488-1 35637307 PMC9184281

[DEV200976C36] Naruse, M., Ono, R., Irie, M., Nakamura, K., Furuse, T., Hino, T., Oda, K., Kashimura, M., Yamada, I., Wakana, S. et al. (2014). *Sirh7/Ldoc1* knockout mice exhibit placental P4 overproduction and delayed parturition. *Development* 141, 4763-4771. 10.1242/dev.114520 25468940 PMC4299276

[DEV200976C37] Norris, G. T. and Kipnis, J. (2018). Immune cells and CNS physiology: Microglia and beyond. *J. Exp. Med.* 216, 60-70. 10.1084/jem.20180199 30504438 PMC6314530

[DEV200976C38] Ono, R., Nakamura, K., Inoue, K., Naruse, M., Usami, T., Wakisaka-Saito, N., Hino, T., Suzuki-Migishima, R., Ogonuki, N., Miki, H. et al. (2006). Deletion of *Peg10*, an imprinted gene acquired from a retrotransposon, causes early embryonic lethality. *Nat. Genet.* 38, 101-106. 10.1038/ng1699 16341224

[DEV200976C39] Pandya, N. J., Wang, C., Costa, V., Lopatta, P., Meier, S., Zampeta, F. I., Punt, A. M., Mientjes, E., Grossen, P., Distler, T. et al. (2021). Secreted retrovirus-like GAG-domain-containing protein PEG10 is regulated by UBE3A and is involved in Angelman syndrome pathophysiology. *Cell Rep. Med.* 2, 100360. 10.1016/j.xcrm.2021.100360 34467244 PMC8385294

[DEV200976C40] Pastuzyn, E. D., Day, C. E., Kearns, R. B., Kyrke-Smith, M., Taibi, A. V., McCormick, J., Yoder, N., Belnap, D. M., Eriendsson, S., Morado, D. R. et al. (2018). The neuronal gene *Arc* encodes a repurposed retrotransposon Gag protein that mediates intercellular RNA transfer. *Cell* 172, 275-288. 10.1016/j.cell.2017.12.024 29328916 PMC5884693

[DEV200976C41] Peterson, P. K., Gekker, G., Hu, S., Anderson, W. R., Teichert, M., Chao, C. C. and Molitor, T. W. (1996). Multinucleated giant cell formation of swine microglia induced by *Mycobacterium bovis*. *J. Infect. Dis.* 173, 1194-1201. 10.1093/infdis/173.5.1194 8627072

[DEV200976C42] Reemst, K., Noctor, S. C., Lucassen, P. J. and Hol, E. M. (2016). The indispensable roles of microglia and astrocytes during brain development. *Front. Hum. Neurosci.* 10, 566. 10.3389/fnhum.2016.00566 27877121 PMC5099170

[DEV200976C43] Sato, M., Yasuoka, Y., Kodama, H., Watanabe, T., Miyazaki, J.-I. and Kimura, M. (2000). New approach to cell lineage analysis in mammals using the Cre-loxP system. *Mol. Reprod. Dev.* 56, 34-44. 10.1002/(SICI)1098-2795(200005)56:1<34::AID-MRD5>3.0.CO;2-M 10737965

[DEV200976C44] Segel, M., Lash, B., Song, J., Ladha, A., Liu, C. C., Jin, X., Mekhedov, S. L., Macrae, R. K., Koonin, E. V. and Zhang, F. (2021). Mammalian retrovirus-like protein PEG10 packages its own mRNA and can be pseudotyped for mRNA delivery. *Science* 373, 882-889. 10.1126/science.abg6155 34413232 PMC8431961

[DEV200976C45] Sekita, Y., Wagatsuma, H., Nakamura, K., Ono, R., Kagami, M., Wakisaka, N., Hino, T., Suzuki-Migishima, R., Kohda, T., Ogura, A. et al. (2008). Role of retrotransposon-derived imprinted gene, *Rtl1*, in the feto-maternal interface of mouse placenta. *Nat. Genet.* 40, 243-248. 10.1038/ng.2007.51 18176565

[DEV200976C46] Shi, J., Zhao, Y., Wang, Y., Gao, W., Ding, J., Li, P., Hu, L. and Shao, F. (2014). Inflammatory caspases are innate immune receptors for intracellular LPS. *Nature* 514, 187-192. 10.1038/nature13683 25119034

[DEV200976C47] Sierra, A., Encinas, J. M., Deudero, J. J. P., Chancey, J. H., Enikolopov, G., Overstreet-Wadiche, L. S., Tsirka, S. E. and Maletic-Savatic, M. (2010). Microglia shape adult hippocampal neurogenesis through apoptosis-coupled phagocytosis. *Cell Stem Cell* 7, 483-495. 10.1016/j.stem.2010.08.014 20887954 PMC4008496

[DEV200976C48] Song, S. U., Gerasimova, T., Kurkulos, M., Boeke, J. D. and Corces, V. G. (1994). An Env-like protein encoded by a Drosophila retroelement: evidence that gypsy is an infectious retrovirus. *Genes Dev.* 8, 2046-2057. 10.1101/gad.8.17.2046 7958877

[DEV200976C49] Suyama, M., Torrents, D. and Bork, P. (2006). PAL2NAL: robust conversion of protein sequence alignments into the corresponding codon alignments. *Nucleic Acids Res.* 34, W609-W612. 10.1093/nar/gkl315 16845082 PMC1538804

[DEV200976C50] Utans, U., Arceci, R. J., Yamashita, Y. and Russell, M. E. (1995). Cloning and characterization of allograft inflammatory factor-1: a novel macrophage factor identified in rat cardiac allografts with chronic rejection. *J. Clin. Invest.* 95, 2954-2962. 10.1172/JCI118003 7769138 PMC295984

[DEV200976C51] Xu, B. and Yang, Z. (2013). PAMLX: a graphical user interface for PAML. *Mol. Biol. Evol.* 30, 2723-2724. 10.1093/molbev/mst179 24105918

[DEV200976C52] Yagi, T., Tokunaga, T., Furuta, Y., Noda, S., Yoshida, M., Tsukada, T., Saga, Y., Takeda, N., Ikawa, Y. and Aiwasa, S. (1993). A novel ES cell line, TT2, with high germline-differentiating potency. *Anal. Biochem.* 214, 70-76. 8250257 10.1006/abio.1993.1458

[DEV200976C53] Youngson, N. A., Kocialkowski, S., Peel, N. and Ferguson-Smith, A. C. (2005). A small family of sushi-class retrotransposon-derived genes in mammals and their relation to genomic imprinting. *J. Mol. Evol.* 61, 481-490. 10.1007/s00239-004-0332-0 16155747

[DEV200976C54] Zhang, L. and Li, W.-H. (2004). Mammalian housekeeping genes evolve more slowly than tissue-specific genes. *Mol. Biol. Evol.* 21, 236-239. 10.1093/molbev/msh010 14595094

