# Supplementary material for: Retrovirus-derived RTL5 and RTL6 genes are novel constituents of the innate immune system in the eutherian brain
Source: Development. 2022 Sep 27;149(18):dev200976. doi: 10.1242/dev.200976 (PMC9641642; doi:10.1242/dev.200976)
Supplement: Supplementary information [file develop-149-200976-s1.pdf]

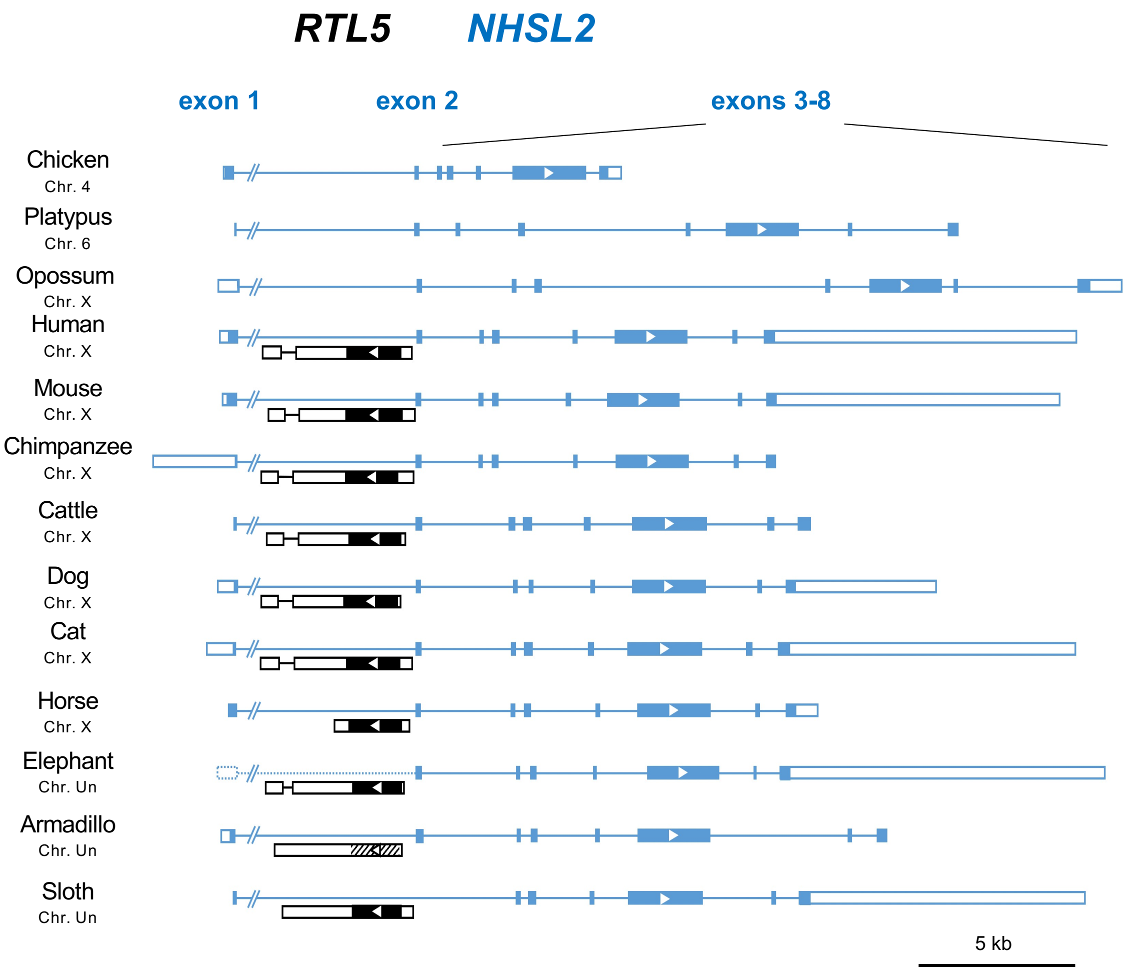

Fig. S1. The chromosomal locations of *RTL5*

The chromosomal locations of *RTL5* in the intron 1 of Nance-Horan syndrome like 2 (*NHSL2*) in the opposite direction. There are no orthologs in chicken (birds), platypus (monotremes) and opossum (marsupials). We obtained the *NHSL2* genomic sequences from the NCBI database and Ensemble. The sequences used for analysis were as follows: Chicken (*Gallus gallus*): bGalGal1.mat.broiler.GRCg7b, XP_015133857.1; Platypus (*Ornithorhynchus anatinus*): mOrnAna1.pri.v4, XP_028923216.1; Opossum (*Monodelphis domestica*): MonDom5, XP_007507794.1; Mouse (*Mus musculus*): GRCm39, XP_011245801.1; Human (Homo sapiens): GRCh38.p13, NP_001013649.2; Chimpanzee (*Pan troglodytes*): Clint_PTRv2, XP_016798720.2; Dog (*Canis lupus familiaris*): ROS_Cfam_1.0, XP_038306208.1; Cat (Felis catus): Felis_catus_9.0, XP_019679523.1; African savanna elephant (*Loxodonta Africana*): Loxafr3.0, XP_023407160.1; Armadillo (*Dasypus novemcinctus*) Dasnov3.0, XP_023439655.1 +XP_023439656.1; Sloth (*Choloepus didactylus*): mChoDid1.pri, XP_037677403.1.

Development • Supplementary information

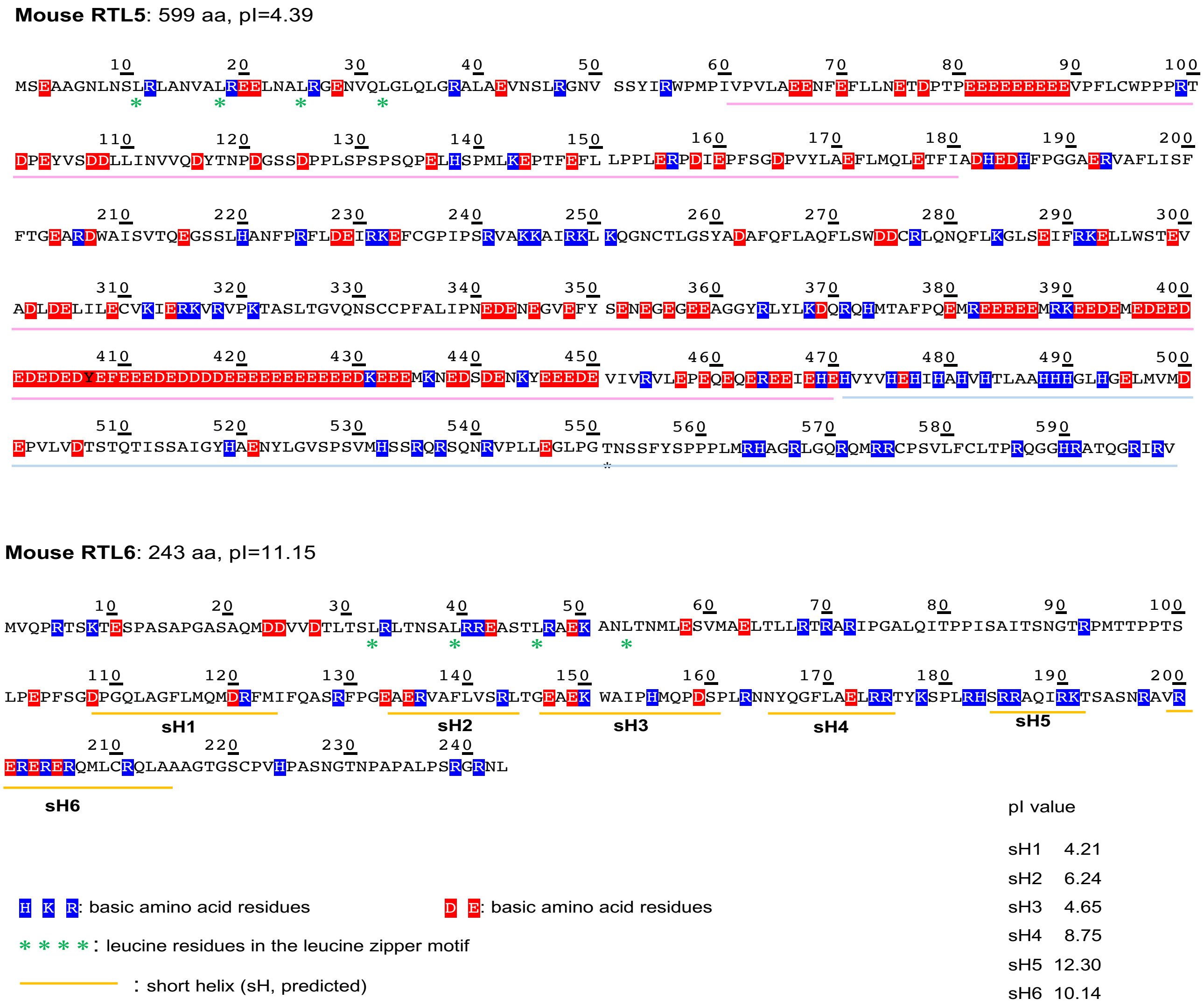

Fig. S2. Amino acid sequences of the mouse RTL5 and RTL6 proteins

Amino acid (aa) sequences are presented along with the number and position of their basic and acidic aa residues. **(Top)** The mouse RTL5 protein contains 72 basic and 137 acidic aa residues out of a total of 599 aa, and so is strongly acidic (pI=4.39), especially the two regions underlined in pink, from 61 to 180 aa (a total of 120 aa: pI=3.55) and from 301 to 470 aa (a total of 170 aa: pI=3.82). However, they have also a basic part underlined in light blue, from 471 to 599 aa (a total of 129 aa: pI=10.02) in the C-terminus. **(Bottom)** The mouse RTL6 protein contains 34 basic and 20 acidic aa residues out of a total of 243 aa, and thus is extremely basic (pI=11.15). In particular, the basic aa residues are concentrated in the region from the last part of short helix (sH) 4 to the C-terminus including sH5 and sH6 (pI=12.30 and 10.14, respectively) estimated by the 3D structure of the sushi-ichi GAG protein according to the SWISS-MODEL prediction (Bienert, et al., 2017).

Development • Supplementary information

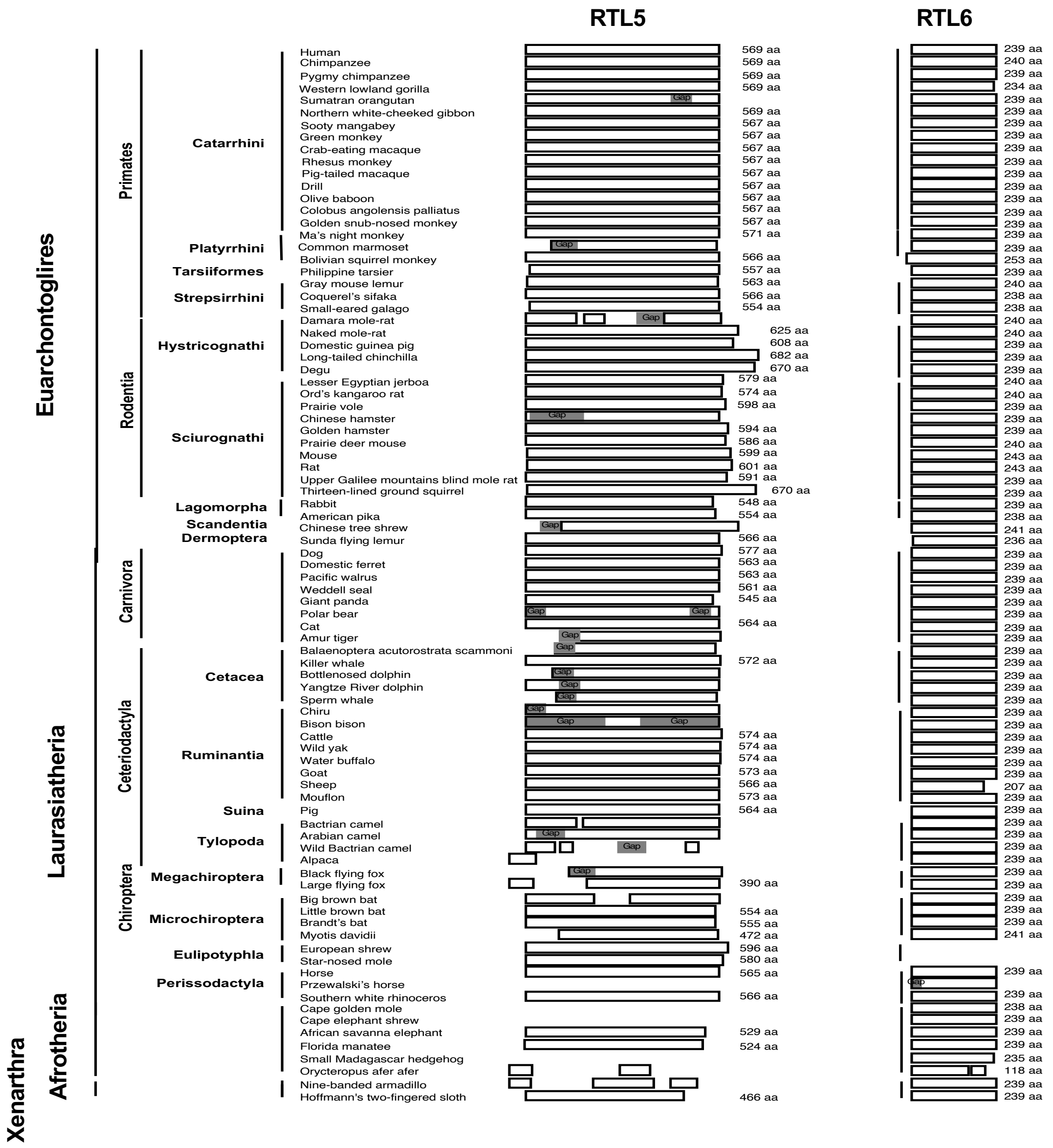

Fig. S3. Conservation of the RTL5 and RTL6 proteins in eutherian species

(Left) *RTL5* has been confirmed in 82 out of 86 eutherian species. It is likely that *RTL5* has become dysfunctional in certain eutherian species. There is no data published on the Prairie vole, Przewaiski's horse, Cape golden mole or Cape elephant shrew. The RTL5 ORF seems disrupted by mutations in 8 species, such as Damara mole-rat, Bactrian camel, wild Bactrian camel, alpaca, large flying fox, big brown bat, *Orycteropus afer afer* and nine-banded armadillo. Sixteen species have small or large sequence gaps in the RTL5 ORF. **(Right)** *RTL6* was confirmed in 84 out of 86 eutherian species. It comprises mostly from 239 to 243 amino acids with two notable exceptions, sheep (207 aa) and *Orycteropus afer afer* (118 aa). It is possible that the latter two are sequencing errors. In the case of Przewaiski's horse, there is a sequence gap in its N-terminus, however, the remaining part is 100% homologous to horse, therefore, it is likely that Przewaiski's horse RTL6 has 239 aa. No data in European threw and star-nosed mole.

Development • Supplementary information

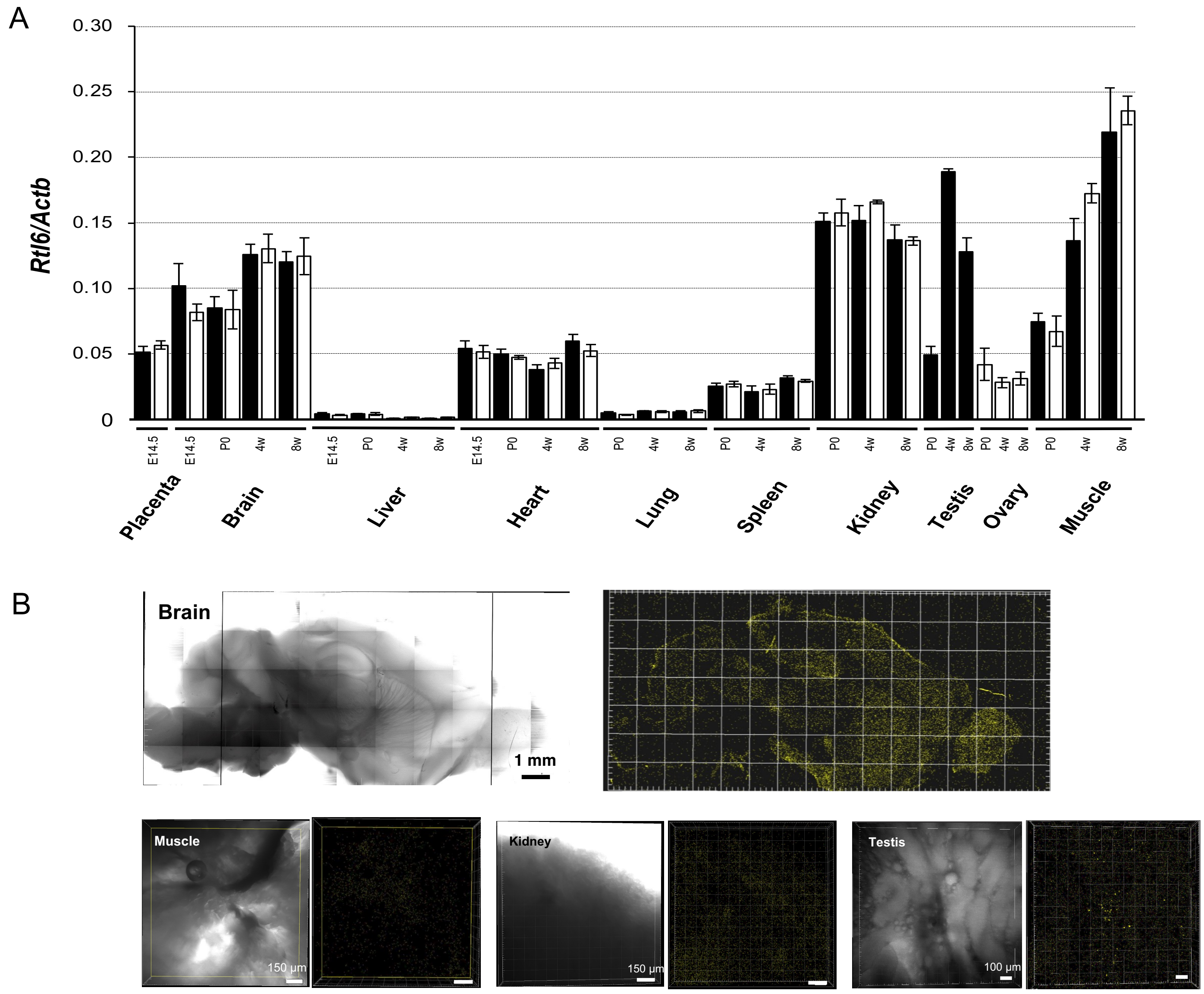

Fig. S4. The expression levels of *Rtl6* mRNA and RTL6-CV protein

(A) *Rtl6* mRNA expression level in several organs and tissues during development. *Rtl6* mRNA expression in the muscle, kidney and testis is higher than that in the brain during development and growth. Black and white bars: male and female samples, respectively (N=3). **(B)** The Venus signals in the brain (top) in 6w adults (N=4) and hindlimb muscle (bottom left), kidney (bottom middle) and testis (bottom right) in 4w adult (N=2). Transmission (left) and Venus signal images (right) are shown in each column. In the latter three, no Venus signals were detected in the top range of ACE9 signals. However, in the testis, a few Venus-positive large granules (5 µm in diameter) were observed in the seminiferous tubules.

Development • Supplementary information

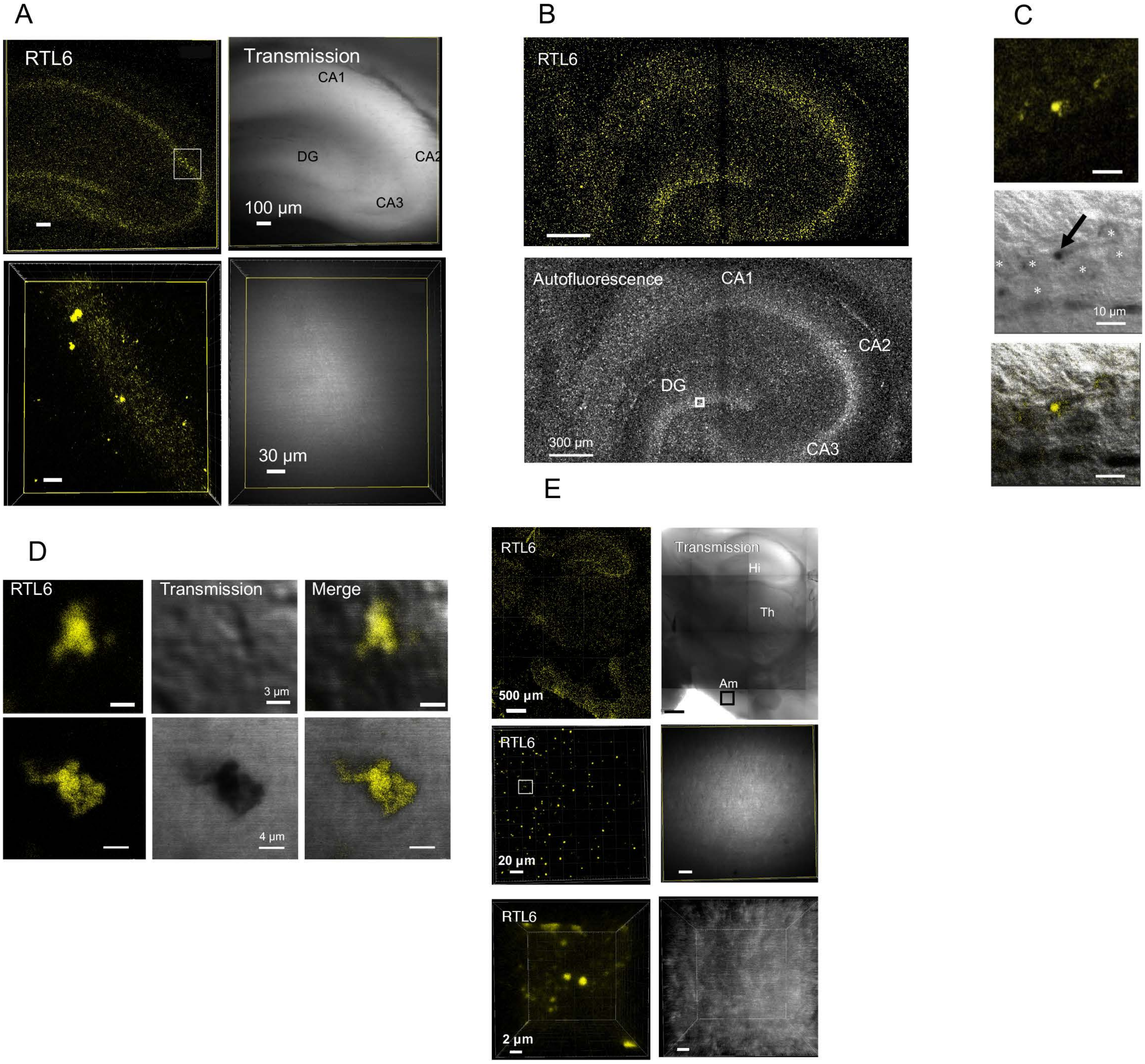

Fig. S5. Venus-positive cells in hypothalamus and amygdala (A) Venus-positive dots accumulated in the CA1-3 and DG areas of hippocampus in neonate (3w) and adult (6w). Top: the dorsal part of the 6w hippocampus. Second: enlarged pictures of the CA2 region indicated as the white square in the top. **(B)** Neonatal hippocampus (3w). Top: Venus signals. Bottom: An autofluorescence image. CA1-3: cornu ammonis subregions 1-3. DG: dentate gyrus. **(C)** Highly magnified images of the DG region of 3w hippocampus (the white square in (B)) showing granule cells extending their dendrites in an upper left direction (nuclei of granule cells are indicated by asterisks). The arrow indicates a Venus-positive cell. **(D)** Venus positive cells in the DG region of 6w hippocampus. Their shapes are also recognizable in the transmission images, indicating that the RTL6-Venus fusion protein is expressed in cytoplasm. **(E)** Top: the posterior half of the 6w brain. Middle: adult amygdala (6w) (enlarged 3D pictures of the black square in the top right figure). Bottom: enlarged 3D pictures of the white square in the left panel of the middle row showing Venus-positive large granules in the amygdala region. Am: amygdala, Hi: hippocampus. Th: thalamus.

Development • Supplementary information

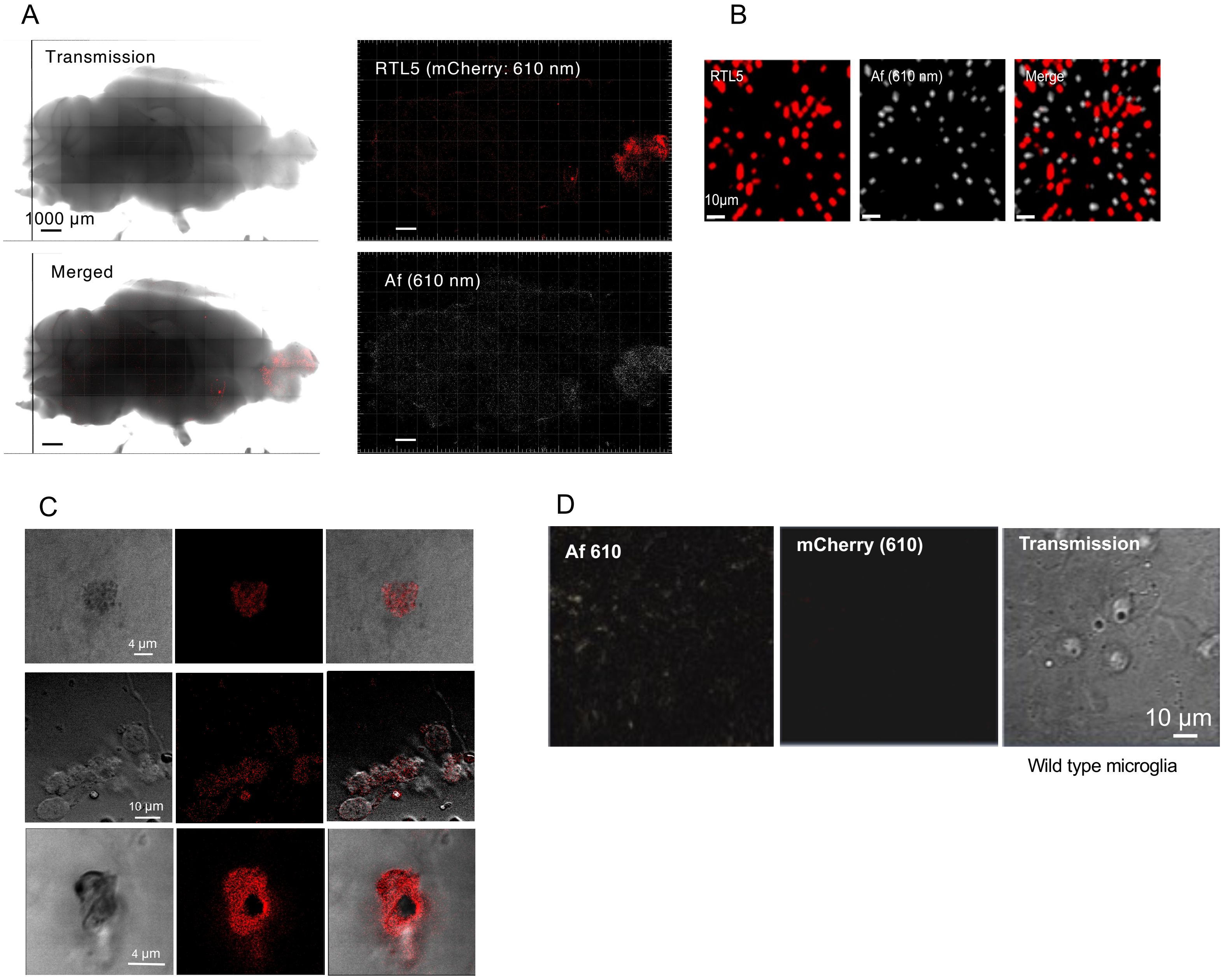

Fig. S6. RTL5-mCherry expression (A) RTL5 expression on the inner surface of the hemispheres in 4w adults (N=2). The pictures of mCherry (top, 610 nm, red signals) and Af610nm (bottom, white signals) are presented. **(B)** Enlarged views of the olfactory bulb. Most of the mCherry-derived red signals were not merged with the Af610nm-derived white signals, indicating they recognized different substances. **(C)** mCherry-positive cells in the olfactory bulb (top), cerebral cortex (middle) and cerebellum (bottom). (D) A population of wild type microglia. Left: Af610nm (white) signals. Center: mCherry (red, 610 nm) signals. Right: a transmission image.

Development • Supplementary information

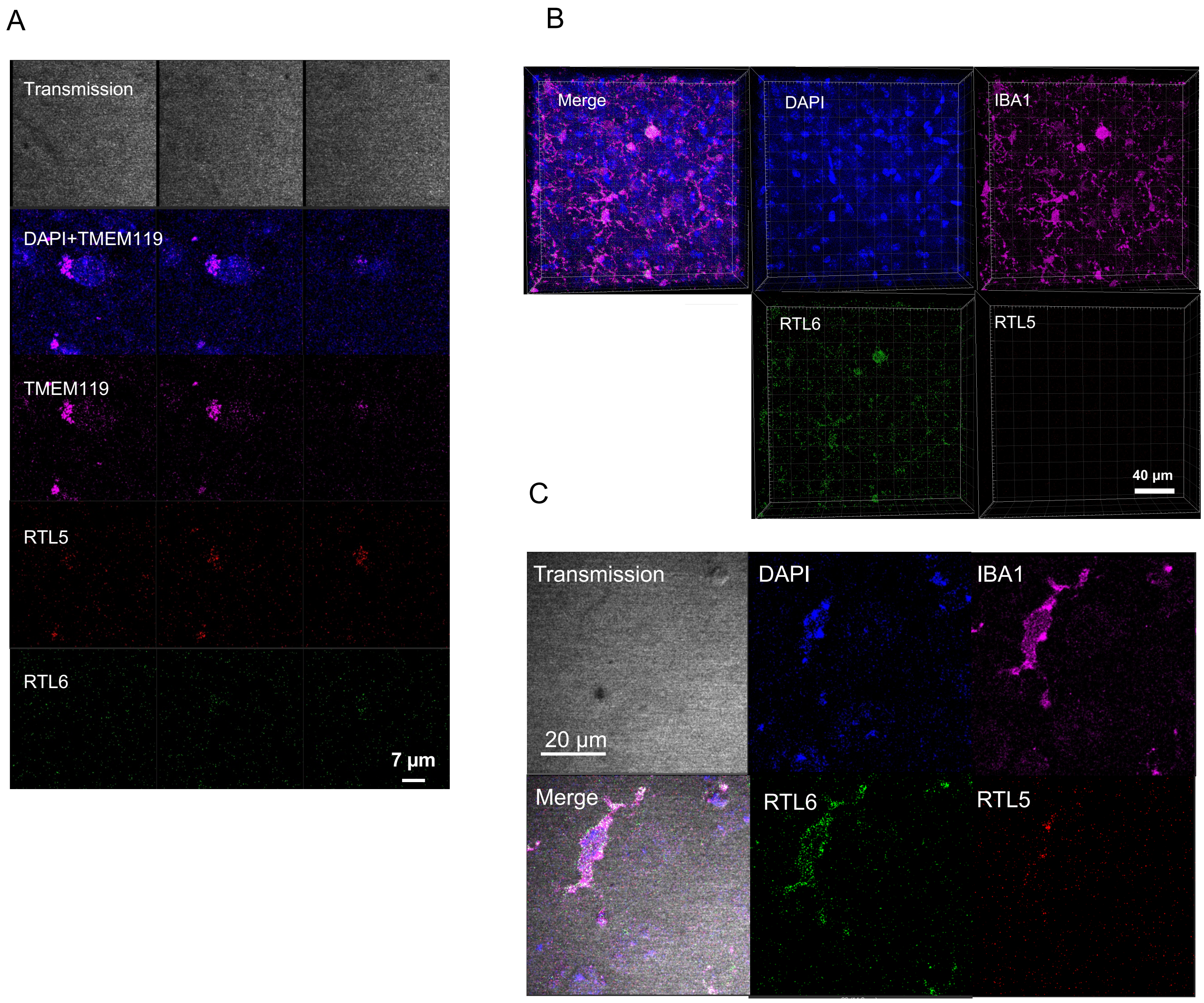

Fig. S7. TMEM119+ and Iba1+ microglia (A) Another example of round type of microglia expressing RTL5 (see Fig. 5D). **(B)** RTL5 expression was hardly detected in Iba1+ ramified microglia. **(C)** RTL5 expression was only detected as a few granules in Iba1+ elongated microglia. Blue, magenta, green and red represent 4', 6-diamidino-2-phenylindole (DAPI), Iba1, RTL6 and RTL5, respectively. Each sequence of photographs at 0.5 µm intervals were presented.

Development • Supplementary information

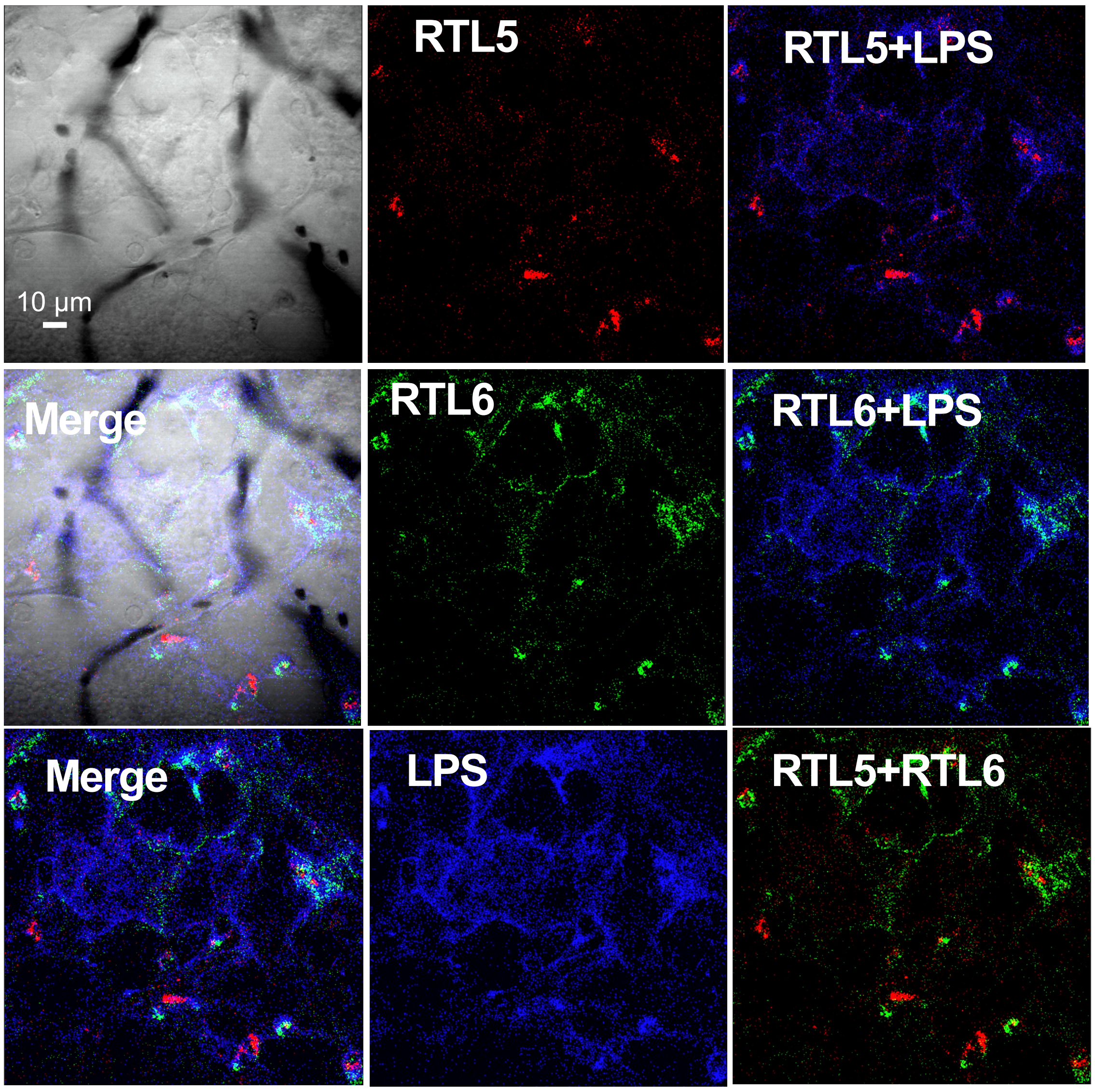

Fig. S8. Giant flattened cells along with the blood capillaries in the cerebral cortex (a lower magnification image of Fig. 5c) LPS was incorporated along the cellular edges where RTL6 had accumulated on the cytoplasmic side in the flattened microglia

Development • Supplementary information

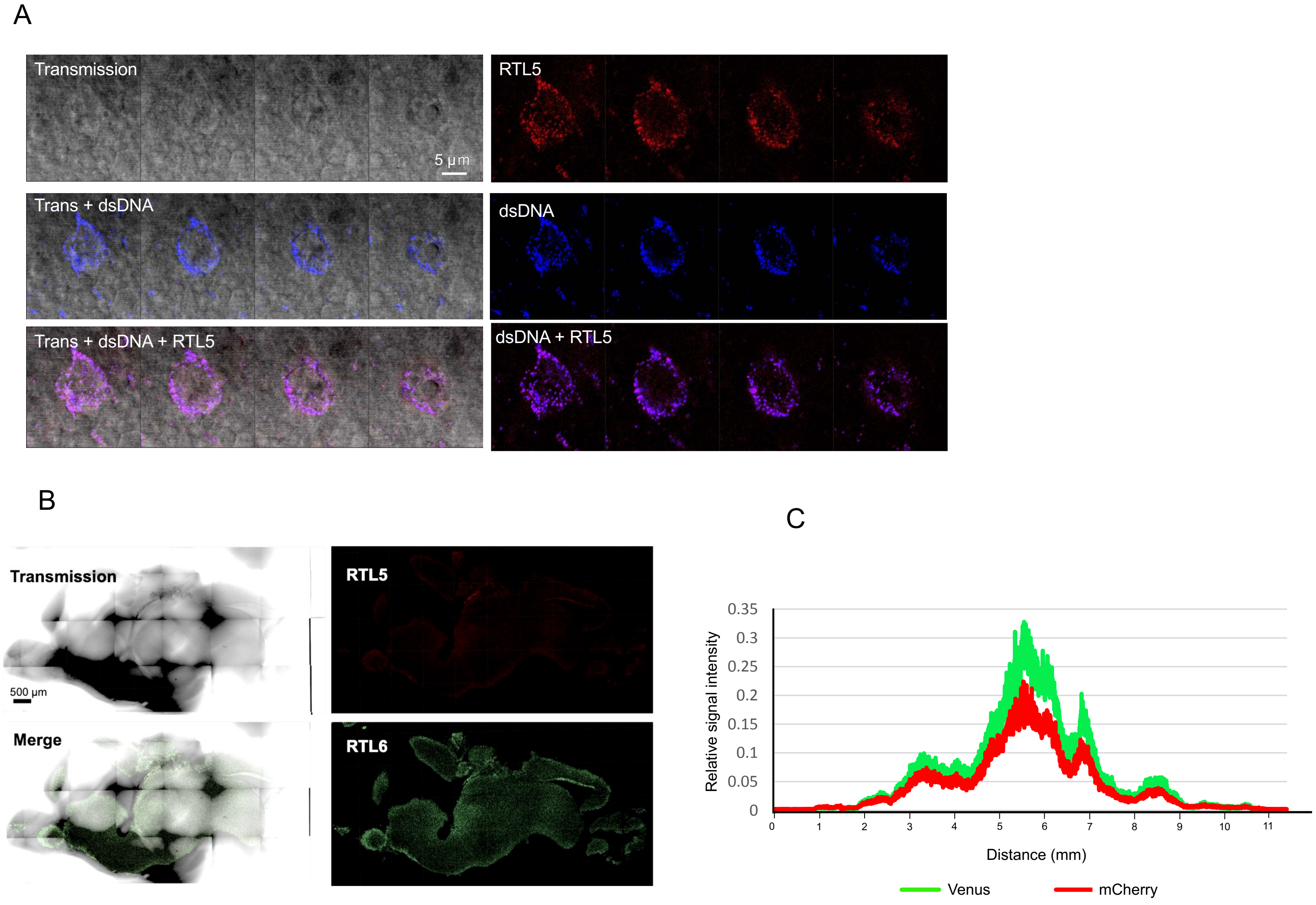

Fig. S9. Location of RTL5-DNA complex around nucleus and non-injection control brain

(A) A round cell similar to TMEM119 positive microglia with accumulated RTL5/dsDNA complexes near nucleus. Transmission (left) and fluorescent images (right) of each RTL5-mCherry (red) and dsDNA (blue) signal are presented as a sequence of photographs at 0.5 µm intervals. **(B)** RTL5 and RTL6 expression in non-injection control mouse (*Rtl5*-mCherry, *Rtl6*-Venus double KI (DKI) mouse (P3 neonate)) (N=3). Transmission (top left), fluorescent images of RTL5 (red, top right) and RTL6 (green, bottom right) and merged image (bottom left), respectively. **(C)** Relative signal intensity of RTL5 and RTL6. In every part of the brain, the relative signal intensity of RTL6-Venus (green) was greater than that of RTL5-mCherry (red) in DKI brain (non-injection control). Due to the methodological limitation in obtaining the absolute value of each signal intensity, it is not possible to compare the signal intensity between different samples, however, possible to compare the relative intensity of each signal in the same brain samples.

Development • Supplementary information

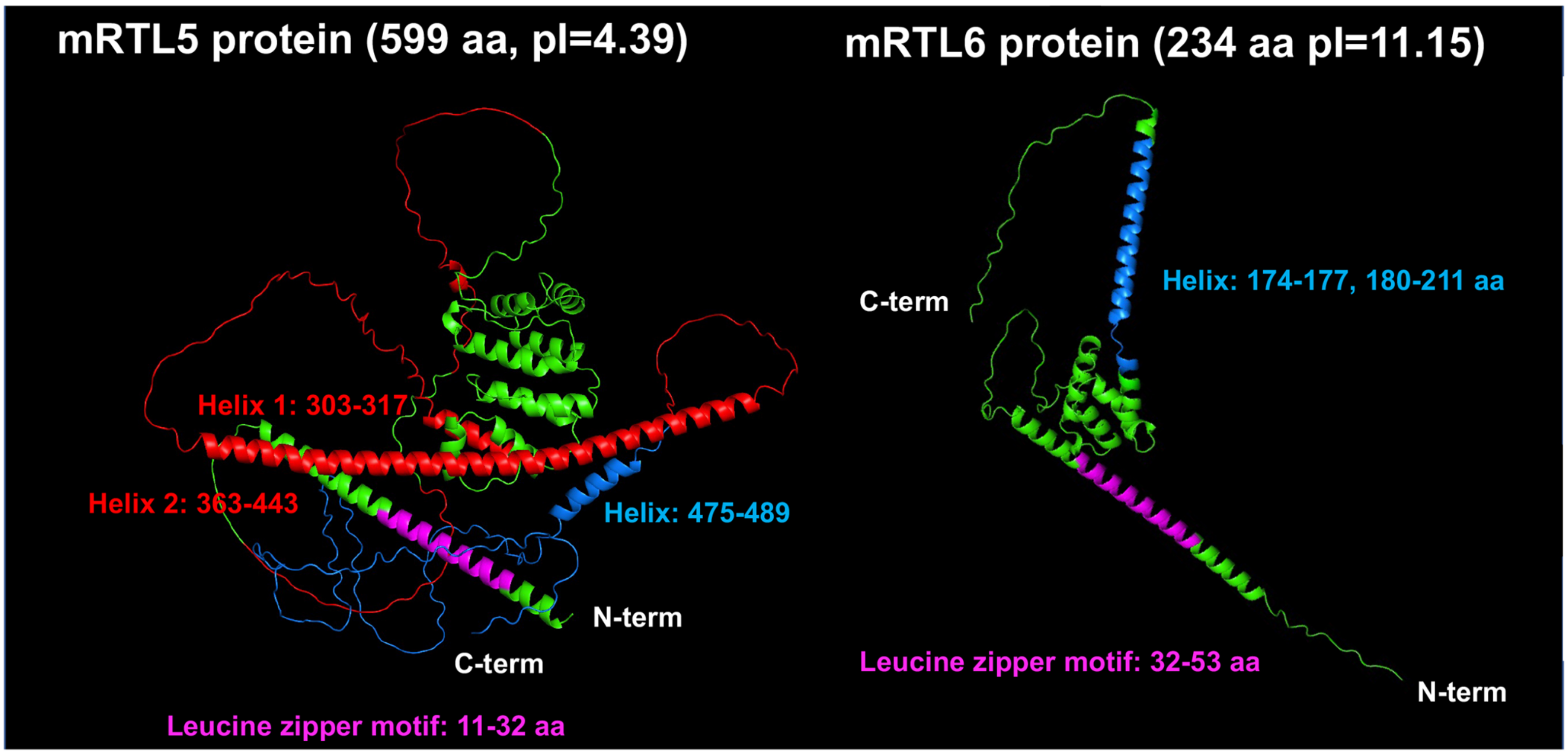

Fig. S10. ColabFold prediction of RTL5 and RTL6 protein's 3D structure

According to ColabFold prediction (Jumper et al., 2021), RTL5 and RTL6 protein's 3D structure was presented. Leucine zipper motifs, acidic and basic parts indicated in Fig. S1C are presented as magenta, red and light blue, respectively. The acidic and basic parts including helix structures are spatially differentiated in the RTL5 protein (left). In the RTL6 protein, the region containing sH5 (184-191 aa) and sH6 (200-214 aa) in Fig. S1C estimated by the sushi-ich GAG (SWISS-MODEL) (Bienert, et al., 2017) form a long helix (180-211 aa) with extremely basic nature.

Additional reference

Bienert, S., Waterhouse, A., de Beer, T. A. P., Tauriello, G., Studer, G., Bordoli, L. and Schwede, T. (2017). The SWISS-MODEL Repository - new features and functionality. *Nucleic Acids Res.* **45**, D313-319.

Development • Supplementary information

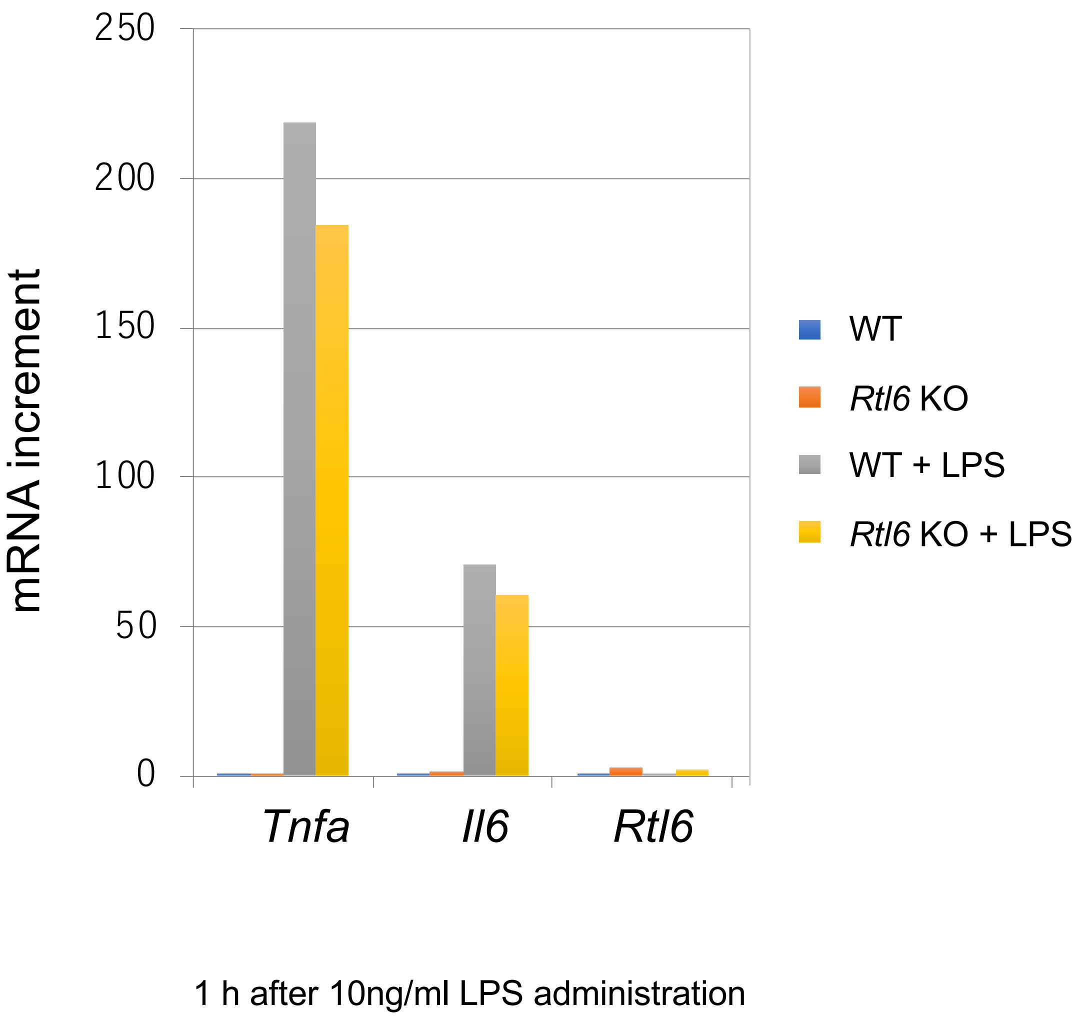

Fig. S11. LPS induction of *Tnfa*, *Il6* and *Rtl6* mRNAs

LPS response was assayed using microglia cells in mixed glia culture. In both WT and *Rtl6* KO microglia *Tnfa* and *Il6* were upregulated 1hr after 10 ng/ml of LPS administration while no *Rtl6* induction was observed (WT: N=4, *Rtl6* KO: N=1).

Development • Supplementary information
