# Supplementary material for: Retrovirus-derived RTL5 and RTL6 genes are novel constituents of the innate immune system in the eutherian brain
Source: Development. 2022 Sep 27;149(18):dev200976. doi: 10.1242/dev.200976 (PMC9641642; doi:10.1242/dev.200976)
Supplement: Supplementary file 1 [file develop-149-200976_review_history.pdf]

Retrovirus-derived *RTL5* and *RTL6* genes are novel constituents of the innate immune system in the eutherian brain

Masahito Irie, Johbu Itoh, Ayumi Matsuzawa, Masahito Ikawa, Hiroshi Kiyonari, Miho Kihara, Toru Suzuki, Yuichi Hiraoka, Fumitoshi Ishino and Tomoko Kaneko-Ishino
DOI: 10.1242/dev.200976

Editor: Haruhiko Koseki

Review timeline

Original submission:	23 May 2022
Editorial decision:	24 June 2022
First revision received:	21 July 2022
Accepted:	14 August 2022

Original submission

First decision letter

MS ID#: DEVELOP/2022/200976

MS TITLE: Retrovirus-derived RTL5 and RTL6 genes are novel constituents of the innate immune system in the eutherian brain

AUTHORS: Masahito Irie, Johbu Itoh, Ayumi Matsuzawa, Masahito Ikawa, Hiroshi Kiyonari, Miho Kihara, Toru Suzuki, Yuichi Hiraoka, Fumitoshi Ishino, and Tomoko Kaneko-Ishino

I have now received all the referees reports on the above manuscript, and have reached a decision. The referees' comments are appended below, or you can access them online: please go to BenchPress and click on the 'Manuscripts with Decisions' queue in the Author Area.

The overall evaluation is positive and we would like to publish a revised manuscript in Development, provided that the referees' comments can be satisfactorily addressed. Please attend to all of the reviewers' comments in your revised manuscript and detail them in your point-by-point response. If you do not agree with any of their criticisms or suggestions explain clearly why this is so. If it would be helpful, you are welcome to contact us to discuss your revision in greater detail. Please send us a point-by-point response indicating your plans for addressing the referee’s comments, and we will look over this and provide further guidance.

Reviewer 1

Advance summary and potential significance to field

It was previously demonstrated that microglia are derived from the extra-embryonic tissues, yolk sac, and that microglia express several TLR proteins such as TLR3, TLR4, and TLR9 for dsRNA, LPS, and non-methylated CpG DNA, respectively.
In this manuscript, two retrovirus-derived RTL5 and RTL6, expressed in the microglia, can also contribute to the innate immunuity of the microglia.
Endogenous retroviruses has been researched in placental development. This manuscript changes its paradigm so that endogenous retroviruses need to be studied in not only placental development but also brain development in the eutherians.

© 2022. Published by The Company of Biologists under the terms of the Creative Commons Attribution License (https://creativecommons.org/licenses/by/4.0/).

Comments for the author

A manuscript entitled "Retrovirus-derived RTL5 and RTL6 genes are novel constituents of the innate immune system in the eutherian brain" by Irie et al., was submitted for its consideration of publication in Development. This manuscript is based upon the data meticulously generated through both loss and gain of function experiments with retrovirus-derived RTL5 and RTL6, examining these retroviral functions on microglia's innate immunity.
It was previously demonstrated that microglia are derived from the extra-embryonic tissues, yolk sac, and that microglia express several TLR proteins such as TLR3, TLR4, and TLR9 for dsRNA, LPS, and non-methylated CpG DNA respectively.

In this manuscript, the authors demonstrated that although the expression of RTL5 and RTL6 transcripts in yolk sac are minute, they become apparent on E9 and definitive expression appears in forebrain and midbrain on E13.5 and P0, the later of which is important for neurogenesis. The presence of RTL6-venus protein in the microglia is found through merging with a microglial marker Iba1. The expression and localization studies are followed by functional studies in which LPS or dsRNA was administered into the brain.

It is very interesting to see that RTL5 and RTL6 are eutherian specific, and that together with the intrinsic ability of innate immunity in microglia, RTL5 and RTL6 play a supportive role in innate immunity of microglia. These redundant pathways could have been required for eutherian brain development, particularly neurogenesis and/or the generation of neural networks.

These findings undoubtedly open new research fields for not only retrotransposon-endogenous retroviruses but also brain neurogenesis/development.
It appeared that both RTL5 and RTL6 proteins are required for the aggregation of unwanted or damaged products. This also suggests that RTL5 and RTL6 play a supportive role on the microglia's innate immunity. The question is whether RTL6 only is sufficient to play a such role or RTL6 requires RTL5 to exert such a role. The next question would be the timing of RTL6 and/or RTL5 insertion to the eutherians, specifically which one of them inserted to the eutherians first and the role the authors found required for one or both insertions to the eutherians.
The model described in Figure 11 is novel and very attractive. This model needs to be described briefly and clearly in Abstract of the revised manuscript. More importantly, sentences as is (lines 377-396) do not explain precisely the importance and novelty of the findings with RTL5 an RTL6, thus rewording is definitively required.
This reviewer does not agree with the way Introduction is written and would like to suggest the following:
The first paragraph should stop at the line 78, ---muscle development (Kitazawa et al., 2020). The authors should then describe about microglia (lines 87-97 of the original manuscript) as the second paragraph, followed by the description of RTL6 and RTL5 as the third paragraph (lines 78-86 of the original manuscript).

Minor comments
The authors should evaluate the use of adverbs in the originally submitted manuscript.
For example (but not limited to);
Line 83; extremely Line 171; only Line 276; deeply
The authors should also evaluate/reevaluate the use of words such as;
Line 287; caltrop, an area denial weapon etc.
The authors should evaluate the use of past tense and/or present tense particularly in the results section.

<u>Reviewer 2</u>

Advance summary and potential significance to field

On this work, Irie and colleagues, report that two retrotransposon gag-like proteins (RTL5 and 6) are coding for microglia proteins involved in brain innate immunity against distinct pathogens. They have created mice with reporter fluorescent genes to visualize their expression in the CNS. The proteins also respond to different stimuli, such as LPS and dsRNA. Moreover, they knockout these genes in mice to study their function.

© 2022. Published by The Company of Biologists under the terms of the Creative Commons Attribution License (https://creativecommons.org/licenses/by/4.0/).

This manuscript has several strengths, it is well-written with a technical tour-de-force, and the story is captivating. It beautifully combines molecular evolution with functional cellular readouts. On the original side this is perhaps the first evidence of eutherian-specific genes acquired from retroviral infection that are now functioning as part of the innate immune system in the eutherian brain. The proposed mechanistic hypothesis for the presence of intra and extracellular proteins is reasonable. I also enjoyed reading their speculation about the origins of these genes in the yolk sac. Finally, they have a down-to-earth discussion about the technical limitations of their work.

Comments for the author

I only have one minor suggestion for the authors. Since they were able to isolate the microglia from these KOs, it shouldn't be too complicated to perform a global gene expression analysis. Such experiment might help to elucidate what are the downstream pathways that are also related to these two genes in microglia. I totally understand that this is a risky experiment and might be outside the scope here.

Reviewer 3

Advance summary and potential significance to field

In the manuscript, Irie et al. described their findings on the retroviral two genes, RTL5 and RTL6. They clearly show the localization of the two genes using knock-in mice and fluorescent reporter systems and carefully monitor the reaction of two genes against pathogenic substances. The proposed model that retroviral genes are involved in the immune system via the formation of the complex with pathogens is novel and intriguing.

Comments for the author

This reviewer wants to see more biochemical experimental data on the interaction with LPS and dsRNA. There are several specific comments for the manuscript below.
1) Line123: I could not understand why the RefSeq coordinate strongly suggests that the expression of the mouse RTL6 protein is quite low. Please explain in more detail.
2) Line 259-268: The LPS response in Rtl5 KO mice should be described, or predictable results should be discussed.
3) Line 269-275: Were the chain-like complexes with the dsRNA (Fig 7C) observed in KO mice? In particular, does RTL6 form the chain-like complex in the absence of RTL5? This would be important for understanding the functional connection between the complex formation and dsRNA removal.
4) When examining the in vivo expression of TLRs, there may also be an impact of fusing Venus and mCherry, which should be discussed.

Minor points:
5) Fig. 1: a.a. should be aa to match the abbreviation in the text.
6) Fig. 2B: What is the leftmost lane for?
7) Throughout the manuscript and in the Figures, 3' and 5' should be described as 3' and 5'
8) Fig. S1B should probably be included in Figure 1.
9) Why has RTL5 been lost in some eutherians? Discuss if you can speculate on this in any way.
10) Experiments in inoculating mice with RNA viruses that multiply in the brain e.g. Japanese encephalitis virus, in the brain are relatively easy. It does not have to be done this time, but this reviewer would like to see an infection experiment.

First revision

Author response to reviewers' comments

Responses to the reviewers

We thank all the reviewers for their constructive suggestions and comments.

© 2022. Published by The Company of Biologists under the terms of the Creative Commons Attribution License (https://creativecommons.org/licenses/by/4.0/).

We addressed all the reviewers' comments in the revised manuscript and we are sure that it has been improved a lot by adding new data suggested by the reviewers.

Reviewer 1 Comments for the Author:
A manuscript entitled "Retrovirus-derived RTL5 and RTL6 genes are novel constituents of the innate immune system in the eutherian brain" by Irie et al., was submitted for its consideration of publication in Development. This manuscript is based upon the data meticulously generated through both loss and gain of function experiments with retrovirus-derived RTL5 and RTL6, examining these retroviral functions on microglia's innate immunity.
It was previously demonstrated that microglia are derived from the extra-embryonic tissues, yolk sac, and that microglia express several TLR proteins such as TLR3, TLR4, and TLR9 for dsRNA, LPS, and non- methylated CpG DNA, respectively.
In this manuscript, the authors demonstrated that although the expression of RTL5 and RTL6 transcripts in yolk sac are minute, they become apparent on E9, and definitive expression appears in forebrain and midbrain on E13.5 and P0, the later of which is important for neurogenesis. The presence of RTL6-venus protein in the microglia is found through merging with a microglial marker Iba1. The expression and localization studies are followed by functional studies in which LPS or dsRNA was administered into the brain.
It is very interesting to see that RTL5 and RTL6 are eutherian specific, and that together with the intrinsic ability of innate immunity in microglia, RTL5 and RTL6 play a supportive role in innate immunity of microglia. These redundant pathways could have been required for eutherian brain development, particularly neurogenesis and/or the generation of neural networks. These findings undoubtedly open new research fields for not only retrotransposon-endogenous retroviruses but also brain neurogenesis/development.

It appeared that both RTL5 and RTL6 proteins are required for the aggregation of unwanted or damaged products. This also suggests that RTL5 and RTL6 play a supportive role on the microglia's innate immunity. The question is whether RTL6 only is sufficient to play a such role or RTL6 requires RTL5 to exert such a role. The next question would be the timing of RTL6 and/or RTL5 insertion to the eutherians, specifically which one of them inserted to the eutherians first and the role the authors found required for one or both insertions to the eutherians.

Thank you very much for your points.
As shown in Fig. 9, the LPS removal was apparently reduced in *Rtl6* KO. We carried out additional experiments on LPS injection to *Rtl5* KO and observed that this reaction was detected almost unaffected. RTL6 accumulated to LPS as granules and seemed to play a substantial role in LPS removal without RTL5. This result suggests that RTL6 has a major role in LPS removal. Therefore, we added a sentence as below:

Line 279: In the case of LPS injection of the *Rtl5* KO mice, the LPS removal activity was essentially unaffected. RTL6 accumulated to LPS in the form of granules in the cytoplasm of microglia and appeared to play a critical role in LPS removal (Fig. 9E), suggesting that RTL6 has a major role in LPS removal without the formation of a RTL5/RTL6/LPS complex.

As shown in Fig. 8B, in *Rtl6* KO, RTL5 seems to react with LPS where the LPS concentration is low without RTL6, we assume that RTL5 also have a role in the LPS removal to a certain degree.

We agree that the timing of RTL6 and/or RTL5 insertion to the eutherians is a very important and interesting issue. However, we don't have enough data to determine which was first, RTL5 or RTL6, at present. What we only know is that both RTL6 and RTL5 are closely related, presumably possess a common ancestral gene, and must have been inserted into a common eutherian ancestor. It is highly likely that they have accumulated multiple mutations independently for their specific functions.
The model described in Figure 11 is novel and very attractive. This model needs to be described briefly and clearly in Abstract of the revised manuscript.

Thank you very much for your comment.
We added a sentence concerning the importance of extraembryonic tissues in the last of abstract and in the introduction as below:

© 2022. Published by The Company of Biologists under the terms of the Creative Commons Attribution License (https://creativecommons.org/licenses/by/4.0/).

Line 55 in the abstract: Finally, we propose a model emphasizing the importance of extraembryonic tissues as the birth place of retrovirus-derived genes.

Line 100, the last sentence in introduction: We also discuss the importance of extraembryonic tissues, such as the placenta and yolk sac, in which retrovirus-derived sequences are suggested to have been incubated for a long period of time, ultimately becoming novel endogenous genes by a series of selection events.

More importantly, sentences as is (lines 377-396) do not explain precisely the importance and novelty of the findings with RTL5 and RTL6, thus rewording is definitively required.

We changed the sentence as blow:

Line 406 in the discussion: Among the 11 *RTLs, RTL5* and *RTL6* are the first examples of genes functioning in yolk sac-derived microglia **and playing roles in the front line of brain innate immune responses against distinct pathogens**.

This reviewer does not agree with the way Introduction is written and would like to suggest the following: The first paragraph should stop at the line 78, ---muscle development (Kitazawa et al., 2020). The authors should then describe about microglia (lines 87-97 of the original manuscript) as the second paragraph, followed by the description of RTL6 and RTL5 as the third paragraph (lines 78-86 of the original manuscript).

We agree with this suggestion and changed the introduction part according to the reviewer's suggestion. We put the description of RTL6 and RTL5 in the third paragraph in the revised version of manuscript followed by additional sentences (bold), but remained the description of the last part of RTL1 and Rtl4, in the first paragraph.

Line 95-103 (the last part of the introduction in revised manuscript): In this work, we address how *RTL6* (aka *SIRH3* or *LDOC1-like* (*LDOC1L*) and the phylogenetically related *RTL5* (aka *SIRH8* or retrotransposon Gag domain like 4 (*RGAG4*)) contribute to the present day eutherian development/growth systems as eutherian-specific, acquired genes, **with *RTL6* being the most conserved of the *RTL* genes in eutherians. Importantly, both *RTL6* and *RTL5* play roles in the innate immune response in the brain against pathogens. We also discuss the importance of extraembryonic tissues, such as the placenta and yolk sac, in which retrovirus-derived sequences are suggested to have been incubated for a long period of time, ultimately becoming novel endogenous genes by a series of selection events.**

Minor comments
The authors should evaluate the use of adverbs in the originally submitted manuscript.

For example (but not limited to);
Line 83; extremely:
Line 98 (in the revised manuscript); with *RTL6* being **the most** conserved of the *RTL* genes in eutherians.

Line 171; only:
We deleted "only" from the lines 171(181), 305 (328) and replaced with "evidently" in the line 277 (297).

Line 276; deeply:
We deleted "deeply" from the line 276 (296) and replaced with "quite" in the line 373 (403).

The authors should also evaluate/reevaluate the use of words such as;
Line 287; caltrop, an area denial weapon etc.

We changed this sentence as below:
Line 304: Both proteins are present as intra- as well as extracellular granules in the brain, so it is possible that they act as an emergency response and immediately trap invaded pathogens in order to prevent them from spreading.

© 2022. Published by The Company of Biologists under the terms of the Creative Commons Attribution License (https://creativecommons.org/licenses/by/4.0/).

The authors should evaluate the use of past tense and/or present tense, particularly in the results section.

Thank you for your suggestion. The entire manuscript was re-edited by a native professional.

Reviewer 2 Comments for the Author:
I only have one minor suggestion for the authors. Since they were able to isolate the microglia from these KOs, it shouldn't be too complicated to perform a global gene expression analysis. Such experiment might help to elucidate what are the downstream pathways that are also related to these two genes in microglia. I totally understand that this is a risky experiment and might be outside the scope here.

We agree with your constructive suggestion. We also thought it was very important. Actually, we have already done the RNAseq experiment using *Rtl6* KO microglia isolated from mixed glial culture. Unexpectedly, what we observed was only approximately 2-fold increment of *Rtl5* mRNA expression (and *Rtl6* itself without ORF), presumably consistent with our results that *Rtl5* has some roles instead of *Rtl6*. However, no other genes exhibited any significant changes. We had almost the same results in the experiment using *Rtl6* KO whole brain although several other genes showed small but substantial change (within 2- fold) in these cases.

Therefore, we assume that interaction between the neurons and microglia in specific brain region(s) is important to elucidate the downstream pathways of RTL6 and also RTL5. Then, we think tha it will be necessary to do the RNAseq analyses using several distinct parts of the brain, such as hypothalamus, midbrain and cortex, independently.

Reviewer 3 Comments for the Author:
This reviewer wants to see more biochemical experimental data on the interaction with LPS and dsRNA. There are several specific comments for the manuscript below.

1) Line123: I could not understand why the RefSeq coordinate strongly suggests that the expression of the mouse RTL6 protein is quite low. Please explain in more detail.

Thank you for your point. There are several reports indicating that the existence of an upstream ORF generally reduces the translational efficiency of downstream ORFs by several mechanisms, such as prevention of re-initiation of ribosome, stalling by encoded peptide and destabilization of mRNA via nonsense-mediated decay (Calvo et al. 2009, Hinnebusch et al. 2016). We added this explanation in the revised manuscript (bold).

Lines 129-134: This strongly suggests that expression of the mouse RTL6 protein is quite low, even if clearly expressed, **because the existence of an upstream ORF generally reduces the translational efficiency of downstream ORFs by a variety of mechanisms, such as prevention of the re-initiation of ribosomes, stalling of encoded peptides and destabilization of mRNA via nonsense-mediated decay (Calvo et al. 2009, Hinnebusch et al. 2016).**

2) Line 259-268: The LPS response in Rtl5 KO mice should be described, or predictable results should be discussed.

Thank you very much for your critical points, (2) and (3). We agree that these are very important issues on RTL5 and RTL6 functions in the pathogen removal reactions.
Therefore, we carried out additional experiments on LPS injection to *Rtl5* KO as shown in Fig. 9E. We observed that the reaction was detected almost unaffected and RTL6 accumulated to LPS as granules and seemed to play a substantial role in LPS removal without RTL5. This suggests that RTL6 is a major player on LPS removal without forming RTL5/RTL6/LPS complex. Therefore, we added a sentence as below:

Lines 279-283: In the case of LPS injection of the *Rtl5* KO mice, the LPS removal activity was essentially unaffected. RTL6 accumulated to LPS in the form of granules in the cytoplasm of microglia and appeared to play a critical role in LPS removal (Fig. 9E), suggesting that RTL6 has a major role in LPS removal without the formation of RTL5/RTL6/LPS complex.

© 2022. Published by The Company of Biologists under the terms of the Creative Commons Attribution License (https://creativecommons.org/licenses/by/4.0/).

3) Line 269-275: Were the chain-like complexes with the dsRNA (Fig 7C) observed in KO mice? In particular, does RTL6 form the chain-like complex in the absence of RTL5? This would be important for understanding the functional connection between the complex formation and dsRNA removal.

We also got a similar result in case of dsRNA injection to *Rtl6* KO. The dsRNA removal was detected almost unaffected, suggesting that RTL5 seems to play substantial role in the dsRNA removal without RTL6. Actually, RTL5 was accumulated to dsRNA as granules in the cytoplasm of microglia (Fig. 10C).
Relating to this question, we also add the data of RTL6 distribution in the dsRNA injected *Rtl5* KO brain. In this case, RTL6 was distributed randomly in the sea of dsRNA (Fig. 10B).

Therefore, we added three sentences as below (Bold):

Lines 287-295: …..while it was unchanged in the *Rtl5* KO mice and remained so even after 110 min when compared to the intensity 25 min after administration (Fig. 10A, middle), indicating that without RTL5 dsRNA removal was significantly delayed in the brain. **RTL6 was dispersed as small dots independently from the distribution of dsRNA analog (Fig. 10B). In the case of dsRNA injection to *Rtl6* KO, the dsRNA removal activity seemed to be unaffected. RTL5 was accumulated to dsRNA as granules in the cytoplasm of microglia (Fig. 10C) but seemed to play substantial role in the dsRNA removal without the formation of chain-like RTL5/RTL6/dsRNA complex.**

4) When examining the in vivo expression of TLRs, there may also be an impact of fusing Venus and mCherry, which should be discussed.

Thank you very much your suggestion.
As we discussed in this issue on Line 303-306 in the original manuscript (Lines 317-321 in the revised manuscript) as below.

Line 318-321: However, we cannot exclude the possibility that the Venus and mCherry tagging changes the expression level and localization of the RTL6 and RTL5 proteins to a certain extent, because at present there are no reliable antibodies to RTL6 and RTL5 that would allow confirmation of the findings.

However, we agree that it is not enough and added some more discussions in two parts.

Lines 321-324: In addition, it is reasonable to hypothesize that the efficiency of the RTL6' and RTL5's functions would be affected by the additional C-terminal Venus or mCherry portion because their functions must have been specified in multiple evolutionary selection events over a long period of time, as discussed below.

Lines 349-356: Although the RTL5-RTL6-LPS and RTL5-RTL6-dsRNA complexes may not play essential roles in the LPS and dsRNA removal reactions (Figs. 9E and 10C), this might be an artifact caused by Venus and/or mCherry tagging. It is reasonable that the RTL5 and RTL6 proteins without the Venus and/or mCherry tagging would exhibit more rapid responses and the actual, endogenous complexes would be more efficient at pathogen removal. In order to check this possibility, we will need to develop novel quantitative techniques that can be applied to non-fixed brain samples.

Minor points:

5) Fig. 1: a.a. should be aa to match the abbreviation in the text.
Amended.

6) Fig. 2B: What is the leftmost lane for?
This lane represents another high MW marker, but is not essential in this figure, therefore, deleted.

7) Throughout the manuscript and in the Figures, 3' and 5' should be described as 3′ and 5′.

© 2022. Published by The Company of Biologists under the terms of the Creative Commons Attribution License (https://creativecommons.org/licenses/by/4.0/).

Amended.

8) Fig. S1B should probably be included in Figure 1.
We moved Fig. S1B to Fig. 1C in the revised manuscript according to the reviewer 3's suggestion.

9) Why has RTL5 been lost in some eutherians? Discuss if you can speculate on this in any way.
It is very important question but difficult to answer at the moment because we cannot exclude the possibility of DNA sequence errors and/or lack of enough DNA sequences in these species. In 16 species, there are small or large sequence gaps in similar part of the RTL5 ORF, around the N-terminus, suggesting that the DNA sequence of this part is not easy to read. For example, in the Cetacea, killer whale has complete RTL5 sequences.
Therefore, it is likely that other four whales also possess complete RTL5 although they have gaps at the moment.
However, in 8 species, including two camels, a large and small bat, Orycteropus afer afer and armadillo, there are one or more stop codons. Therefore, it is likely that some of these species, RTL5 function is lost although it still remains that it is due to incorrect DNA sequence because they are minor species.
We think it is possible that some species lost *RTL5* function because *RTL5* is not essential for their survival depending on the environment and/or survival strategy. One such example may be *RTL4* in xenarthra species, both armadillo and sloth lost *RTL4* (*SIRH11/ZCCHC16*) function by multiples of mutations (Irie et al. PLoS Genet 2015) although it is a very important gene in cognitive recognition via noradrenalin pathway (Irie et al. PLoS Genet 2015) and it is known as one of responsible genes in autism spectrum disorders in humans (Lim et al. Neuron 2013). We speculate that *RTL4* is not necessary for these animals to avoid attacks by other animals because their life styles are unique: armadillos have strong shell for protection and sloths do not move in most of their time, thereby, hidden away from other animals.

Experiments in inoculating mice with RNA viruses that multiply in the brain, e.g. Japanese encephalitis virus, in the brain are relatively easy. It does not have to be done this time, but this reviewer would like to see an infection experiment.

We agree that such infection experiments are definitely needed to elucidate in precise vivo function of RTL5 and RTL6. Therefore, we add viral infection in the manuscript as below:

Lines 330-333. In addition, it will be important to evaluate the acute and chronic effects of LPS, dsRNA, non-methylated DNA administration **and/or viral infection** in the *Rtl5* KO and *Rtl6* KO mice using other, less invasive methods to assess their biological significance *in vivo.*

We would like to do these experiments in near future because the animal facilities have been full in COVID-19 experiments at the moment.

Second decision letter

MS ID#: DEVELOP/2022/200976

MS TITLE: Retrovirus-derived RTL5 and RTL6 genes are novel constituents of the innate immune system in the eutherian brain

AUTHORS: Masahito Irie, Johbu Itoh, Ayumi Matsuzawa, Masahito Ikawa, Hiroshi Kiyonari, Miho Kihara, Toru Suzuki, Yuichi Hiraoka, Fumitoshi Ishino, and Tomoko Kaneko-Ishino
ARTICLE TYPE: Research Article

I am happy to tell you that your manuscript has been accepted for publication in Development, pending our standard ethics checks.

© 2022. Published by The Company of Biologists under the terms of the Creative Commons Attribution License (https://creativecommons.org/licenses/by/4.0/).

Reviewer 1

Advance summary and potential significance to field

Retrotransposon Gag-like 5 (RTL5, also known as sushi-ichi-related retrotransposon homolog 8 (SIRH8)) and RTL6 (aka SIRH3) are eutherian-specific genes presumably derived from a retrovirus and phylogenetically related to each other. RTL5 and RTL6 are microglial genes having roles in the front line of innate brain immune response. In fact, these proteins display a rapid response to pathogens such as lipopolysaccharide (LPS), double-stranded (ds) RNA analog and non-methylated CpG DNA, acting both cooperatively and/or independently.
Experiments using Rtl6 or Rtl5 knock-out mice provided additional evidence that RTL6 and RTL5 act as factors against LPS and dsRNA in the brain, respectively.
These data provide the first demonstration that retrovirus-derived genes play a role in the eutherian innate immune system.

Comments for the author

This reviewer believes that the revision was done appropriately, more importantly the revised manuscript describes the importance/novel findings as is. In other words, the data/results are tied better and their relationships-functions are explained well. Thus, this reviewer strongly support this manuscript for publication in Development.

Reviewer 2

Advance summary and potential significance to field

The authors have performed the requested RNAseq on isolated microglia. The preliminary data pointed to a more complex situation, requiring further brain dissection. I agree that these experiments are not necessary for this first manuscript. Thus, in my opinion, the manuscript has improved and should be accepted for publication.

Comments for the author

The authors responded to all concerns raise by the reviewers.

Reviewer 3

Advance summary and potential significance to field

This paper reports new functions for the retroelements RTL5 and RTL6. The physiological functions of various retroelements are becoming better understood and this paper shows that RTL5 and RTL6 may function in an inhibitory manner against RNA viruses. The demonstration of new functions of retroelements has further increased the importance of retroelement research. The publication of this paper in Development is very significant.

Comments for the author

The authors answered my questions very sincerely. I am satisfied with their answers. With this revision, I consider this paper to be publishable.

© 2022. Published by The Company of Biologists under the terms of the Creative Commons Attribution License (https://creativecommons.org/licenses/by/4.0/).
